# The predictive validity of a Brain Care Score for late-life depression and a composite outcome of dementia, stroke, and late-life depression: data from the UK Biobank cohort

**DOI:** 10.3389/fpsyt.2024.1373797

**Published:** 2024-07-23

**Authors:** Sanjula D. Singh, Cyprien A. Rivier, Keren Papier, Zeina Chemali, Leidys Gutierrez-Martinez, Livia Parodi, Ernst Mayerhofer, Jasper Senff, Santiago Clocchiatti-Tuozzo, Courtney Nunley, Amy Newhouse, An Ouyang, M. Brandon Westover, Rudolph E. Tanzi, Ronald M. Lazar, Aleksandra Pikula, Sarah Ibrahim, H. Bart Brouwers, Virginia J. Howard, George Howard, Nirupama Yechoor, Thomas Littlejohns, Kevin N. Sheth, Jonathan Rosand, Gregory Fricchione, Christopher D. Anderson, Guido J. Falcone

**Affiliations:** ^1^ Henry and Allison McCance Center for Brain Health, Massachusetts General Hospital, Boston, MA, United States; ^2^ Department of Neurology, Massachusetts General Hospital, Boston, MA, United States; ^3^ Broad Institute of Massachusetts Institute of Technology (MIT) and Harvard, Cambridge, MA, United States; ^4^ Department of Neurology, Yale School of Medicine, New Haven, CT, United States; ^5^ Yale Center for Brain and Mind Health, New Haven, CT, United States; ^6^ Nuffield Department of Population Health, University of Oxford, Oxford, United Kingdom; ^7^ Division of Neuropsychiatry, Massachusetts General Hospital, Boston, MA, United States; ^8^ Center for Genomic Medicine, Massachusetts General Hospital, Boston, MA, United States; ^9^ Department of Neurology, Brigham and Women’s Hospitall, Boston, MA, United States; ^10^ Department of Neurology, Rudolf Magnus Institute of Neuroscience, University Medical Centre Utrecht, Utrecht, Netherlands; ^11^ Department of Medicine, Massachusetts General Hospital, Boston, MA, United States; ^12^ Department of Neurology, University of Alabama at Birmingham (UAB) Heersink School of Medicine, University of Alabama at Birmingham (UAB) McKnight Brain Institute, Birmingham, AL, United States; ^13^ Jay and Sari Sonshine Centre for Stroke Prevention & Cerebrovascular Brain Health, University Health Network, Krembil Brain Institute, Toronto, ON, Canada; ^14^ Department of Medicine, Division of Neurology, The Temerty Faculty of Medicine at the University of Toronto, Toronto, ON, Canada; ^15^ Program for Health System and Technology Evaluation; Toronto General Hospital Research Institute, Toronto, ON, Canada; ^16^ Institute of Health Policy, Management and Evaluation (IHPME), Dalla Lana School of Public Health; University of Toronto, Toronto, ON, Canada; ^17^ Centre for Advancing Collaborative Healthcare & Education (CACHE), University of Toronto, Toronto, ON, Canada; ^18^ Department of Neurosurgery, Elisabeth TweeSteden Ziekenhuis, Tilburg, Netherlands; ^19^ Department of Biostatistics, School of Public Health, University of Alabama at Birmingham, Birmingham, AL, United States; ^20^ Benson-Henry Institute for Mind Body Medicine, Massachusetts General Hospital, Boston, MA, United States

**Keywords:** depression - epidemiology, prevention, risk factor, brain health, stroke, dementia

## Abstract

**Introduction:**

The 21-point Brain Care Score (BCS) is a novel tool designed to motivate individuals and care providers to take action to reduce the risk of stroke and dementia by encouraging lifestyle changes. Given that late-life depression is increasingly recognized to share risk factors with stroke and dementia, and is an important clinical endpoint for brain health, we tested the hypothesis that a higher BCS is associated with a reduced incidence of future depression. Additionally, we examined its association with a brain health composite outcome comprising stroke, dementia, and late-life depression.

**Methods:**

The BCS was derived from the United Kingdom Biobank baseline evaluation in participants with complete data on BCS items. Associations of BCS with the risk of subsequent incident late-life depression and the composite brain health outcome were estimated using multivariable Cox proportional hazard models. These models were adjusted for age at baseline and sex assigned at birth.

**Results:**

A total of 363,323 participants were included in this analysis, with a median BCS at baseline of 12 (IQR: 11-14). There were 6,628 incident cases of late-life depression during a median follow-up period of 13 years. Each five-point increase in baseline BCS was associated with a 33% lower risk of incident late-life depression (95% CI: 29%-36%) and a 27% lower risk of the incident composite outcome (95% CI: 24%-30%).

**Discussion:**

These data further demonstrate the shared risk factors across depression, dementia, and stroke. The findings suggest that a higher BCS, indicative of healthier lifestyle choices, is significantly associated with a lower incidence of late-life depression and a composite brain health outcome. Additional validation of the BCS is warranted to assess the weighting of its components, its motivational aspects, and its acceptability and adaptability in routine clinical care worldwide.

## Introduction

Late-life depression, dementia and stroke and are amongst the age-related brain diseases with the highest prevalence and incidence worldwide (1). Late-life depression, usually characterized as primary depression occurring in individuals over the age of 60, has significant implications. It not only increases mortality and morbidity rates but also imposes a considerable economic burden, both directly through healthcare costs and indirectly through impacts on productivity and societal roles (2). Moreover, past research has correlated late-life depression with cognitive decline (e.g., decreased pace of information processing, memory disorders and decreased executive function) (3). Clinically, the diagnoses of late-life depression and dementia can be difficult to disentangle. In a study involving approximately 1000 participants from the Framingham Heart Study, those who had experienced depression before the study’s baseline were found to have a 50% increased risk of dementia over a 17-year follow-up period, compared to participants without a history of depression (4). The same is thought to be true for stroke and depression: people with a depression are reported to have a 45% higher risk for stroke and a 25% higher risk of stroke-related mortality compared to those without a depression (5). Shared modifiable risk factors for late-life depression, dementia and stroke have been consistently reported in previous meta-analyses: and include smoking tobacco, nutrition, physical activity and social-emotional determinants of health, all of which are included in the McCance Brain Care Score [BCS] (2, 6–9).

**Figure 1:**
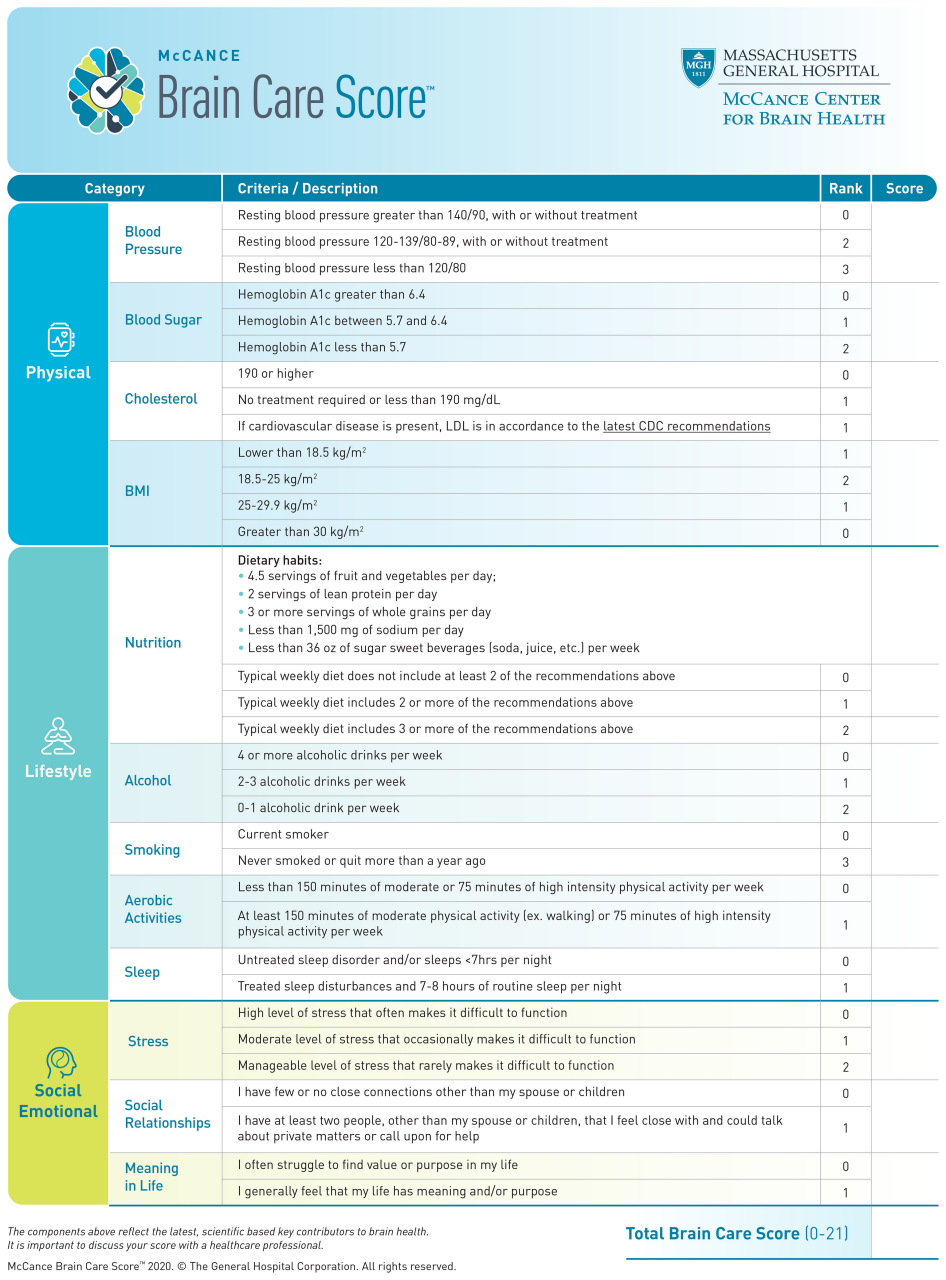
The Brain Care Score.

The aims, characteristics, and development of the BCS have been described elsewhere (10). Briefly, the BCS includes modifiable risk factors for the most common age-related brain diseases (dementia, stroke, and late-life depression), which are endorsed by professional societies and patient advocacy groups (11). The total BCS ranges from 0-21 and consists of 4 physical components (blood pressure, haemoglobin A1c, cholesterol, and Body Mass Index [BMI]), 5 lifestyle elements (nutrition, alcohol intake, smoking, aerobic activities, and sleep), and 3 social emotional factors (stress, relationships, and purpose in life) (**Figure 1**). The BCS is designed to be implemented into routine primary care, ultimately as a motivational tool for health behaviours which stimulate risk factor reduction for dementia, stroke and late-life depression as manifestations of impaired brain health. Previous results have shown clinically relevant and statistically significant associations between the BCS and dementia and stroke incidence using the United Kingdom Biobank (UKB). Herewith, we report the first-ever analyses of associations between the BCS and late-life depression incidence, as well as a combined incidence of stroke, dementia, and late-life depression. Our hypothesis is that a higher baseline BCS in the UKB cohort, which indicates better brain care, would be associated with a lower incidence of late-life depression – in line with the findings for baseline BCS in the UKB and subsequent dementia and stroke incidence (10). Through demonstrating that the Brain Care Score (BCS) serves as a reliable predictor for brain health events, we aim to further substantiate its utility as a clinically relevant tool.

## Methods

### UK Biobank study

The methodology, study design, and inclusion and exclusion criteria of the UK Biobank study, have been delineated in a previous publication (12). In essence, the UK Biobank study constitutes a population-based, prospective cohort study that comprises half a million voluntary participants from the United Kingdom. These participants were enrolled from 22 centres dispersed across the country. Comprehensive data were assembled at baseline (between 2006 and 2010) through questionnaires, anthropometric evaluations, and biomedical measurements. The recruitment targeted individuals aged between 40 and 69 years, with only a negligible number of participants being outside this age range (mainly those who accompanied those invited to the UK Biobank study’s assessment centres). As of the present, there have been three follow-up assessments, conducted in 2012-2013, 2014 onwards, and 2019 onwards, respectively. Health outcomes for all participants are consistently gathered via linkage to health data, encompassing both hospital and mortality data. The UK Biobank study was performed in accordance with the principles established in the Declaration of Helsinki and received approval from the Northwest Multi-Centre Research Ethics Committee (reference number 06/MRE08/65). All human research participants in the UK Biobank study provided their informed consent.

### Exposure: derivation of individual BCS components

The BCS is a three-dimensional instrument that captures physical, lifestyle, and social and emotional measures. Physical measures include blood pressure, blood sugar, cholesterol, and Body Mass Index (BMI); lifestyle measures include nutrition, alcohol consumption, smoking, aerobic activities, and sleep; and emotional measures include stress, social relationships, and meaning in life (**Figure 1**) (13). In the context of the present study, the Brain Care Score (BCS) has been tailored using the data collected by the UK Biobank (UKB), hence leading to a modified version of the BCS (refer to **Table 1**; for detailed variable definitions of the UKB-derived BCS and discrepancies with the original BCS, see **Supplementary Information Table S1**). The process of deriving individual BCS components started with the precise criterion for each component. Adjustments were made only if required due to statistical power considerations or in the absence of relevant data within the UK Biobank. For all components of the BCS that rely on self-reported data, participants who responded with “do not know” or “prefer not to answer” in the UKB questionnaires were omitted from the study (12, 14).

**Table 1:** Brain Care Score in the UK Biobank.

Category		Criteria/description	Rank
**Physical**	**Blood pressure**	Systolic *or* diastolic blood pressure of greater than 140/90 mmHg	0
		Systolic *or* diastolic blood pressure 120–140/80–90 mmHg, and systolic *and* diastolic blood pressure lower than 140/90 mmHg	2
			3
	**Blood Glucose**	Haemoglobin A1c greater than 6.4%	0
		Haemoglobin A1c between 5.7 and 6.4%	1
		Haemoglobin A1c less than 5.7%	2
	**Cholesterol**	Total cholesterol 190 mg/dL or higher	0
		Total cholesterol less than 190 mg/dL	1
	**Body Mass Index**	Lower than 18.5 kg/m^2^	1
		Between 18.5 and 25 kg/m^2^	2
		Higher than 25 and lower than 30 kg/m^2^	1
		Higher than or equal to 30 kg/m^2^	0
**Lifestyle**	**Nutrition**	Dietary habits: • 4.5 or more servings of fruit and vegetables per day • A red meat score of 1 or 2 • 3 or more servings of bread slices or cereal bowls per day • Sometimes, rarely, or never add salt to a meal	
		Typical diet does not include at least 2 of the recommendations above	0
		Typical diet includes 2 of the recommendations above	1
		Typical diet includes 3 or more of the recommendations above	3
	**Alcohol consumption**	Drinking ≥3 times/week	0
		Drinking 1–2 times/week or 1–3 times/month	1
		Drinking only on special occasions or never	2
	**Smoking**	Current smoker	0
		Former or never smoker	2
	**Aerobic activities**	At least 10 minutes of moderate or vigorous activity on fewer than 5 days/week	0
		At least 10 minutes of moderate or vigorous activity on 5 or more days/week	1
	**Sleep**	Less than 7 hours/day	0
		7 or more hours/day	1
**Social Emotional**	**Stress**	Self-perceived tension, fidgetiness, or restlessness several days, more than half the days, or nearly every day in the last 2 weeks	0
		No self-perceived tension, fidgetiness, or restlessness in the last 2 weeks	1
	**Social relationships**	No friends or family members outside the household; no or almost no visits, or only once every few months	0
		Visits once a month, once a week, two to four times a week, or almost daily	1
Total Brain Care Score (0–19)			

The red meat score is based on beef, pork, and lamb/mutton consumption, in which an individual score was first assigned for each meat type (“Never” or “Less than once a week” with 0; “Once a week” or “2–4 times a week” with 1; and “5–6 times a week” or “Once or more daily” with 2); these were then summed, with a score of 1–2 dichotomized into 1 and less than 1 or more than 2 with a 0. Moderate activity includes physical activities such as carrying light loads or cycling; vigorous activity includes activities such as fast cycling, aerobics, or heavy lifting.

The BCS is determined by the summation of the specific scores allocated to each component for an individual. To illustrate: a person who obtains the lowest score in all components will have a BCS of 0, while a person consistently achieving the highest score — either 1, 2, or 3, contingent upon the component — will possess a BCS of 19. Originally, the BCS spanned a range of 0 to 21, but when adapted for the UK Biobank, the scale was adjusted to span from 0 to 19 due to modifications in the scoring for nutrition, stress, and life purpose. A greater BCS correlates with superior brain care. An increase of five points in the total BCS, for example from 0–5 or 10–15, is indicative of a significant, yet attainable, enhancement in one’s brain care. Consequently, a 5-point increment in BCS can be set as an initial target for patients and healthcare providers, with several strategies available to attain this improvement. For instance, a 5-point elevation in BCS could be accomplished through: ceasing smoking and reducing stress levels (no symptoms of tension, restlessness, or anxiety in the past two weeks) while improving social connections (engaging with family or friends at least once monthly); or reducing alcohol intake (from 4 units per week to less than 1 unit per week or only on special occasions), and managing blood pressure (from >140/90 mmHg to <120/80 mmHg); or managing weight (from a BMI ≥30 kg/m2 to a range of 18.5–25 kg/m2) and controlling blood sugar levels (from HbA1c >6.4 to HbA1c <5.7). For the current study, the BCS has been calculated from the data made available by the UKB, and follows the exact same definitions used in prior research (10).

### Outcome assessment: incident late-life depression

The hospital data that is linked with the UK Biobank cohort is derived from Hospital Episode Statistics for England (censoring date: September 30, 2021), the Scottish Morbidity Record (censoring date: July 31, 2021), and the Patient Episode Database for Wales (censoring date: February 28, 2018). The mortality data for England and Wales is provided by NHS Digital (censoring date: September 30, 2021) and the NHS Central Registries, National Records of Scotland (censoring date: October 31, 2021). In accordance with prior research (15, 16), late-life depression cases were derived from multiple ICD-10 codes appearing at or beyond the age of 60. These codes included: depressive episodes (F32), recurrent depressive disorder (F33), persistent mood (affective) disorders (F34), other mood (affective) disorders (F38), or unspecific mood (affective) disorders (F39). Individuals with earlier life depression, defined as a depression ICD-10 code appearing before the age of 60, as well as those whose earlier life depression extended into later life were excluded from the analyses. Additionally, and following the research mentioned previously (15, 16), individuals with any of the following codes were excluded from the analyses: delirium, not induced by alcohol and other psychoactive substances (F05), other mental disorders due to brain damage and dysfunction and physical disease (F06), personality and behavioural disorders due to brain disease, damage and dysfunction (F07), unspecified organic or symptomatic mental disorder (F09), mental and behavioural disorders due to psychoactive substance use (F10-F19), schizophrenia, schizotypal and delusional disorders (F20-29), manic episodes (F30), and bipolar affective disorder (F31). Any primary or secondary diagnosis or a contributory cause of death citing the included ICD codes was considered a late-life depression, except for events that occurred before the baseline measurement or in the first two years of follow-up (which was defined according to the data source listed above). The exclusion of such events was done to address any concerns of reverse causation (17).

### Statistical analyses

#### Complete case analyses

This study considered all UK Biobank participants with fully available BCS data while excluding those with any missing information concerning one or more individual BCS components (the ‘meaning of life’ component was an exception, as it was missing for all UKB participants).

#### Distribution of the BCS components and the total BCS

The distributions of measurements, self-reported responses, and data missing from UKB questionnaires were documented. Further, the distribution of the total BCS of the included UKB participants was presented, and the median along with the interquartile range (IQR) or mean and standard deviation (SD) were reported.

#### Cox proportional hazard regression models

In order to estimate the associations of incident dementia, stroke, and late-life depression with five-point differences in the BCS, we utilized Cox proportional hazard regression models on non-stratified samples. These were adjusted for gender (female versus male) and age at baseline (as a continuous variable) and were separately employed on samples stratified by age group at baseline (<50, 50-59, >59 years), with adjustments made for sex. The time to event for cases was defined as the number of days from the baseline survey to the date of the first occurrence of dementia, stroke or late-life depression. For the other participants, the time to event was identified as the number of days to the censoring date, based on the source of hospital data (as listed above), or, for those who died due to other causes, the date of death. Cox regression models were conducted on one primary outcome: late-life depression, and one secondary outcome: the composite of dementia, stroke or late-life depression. In estimating the per 5-point BCS risk increase for the composite outcome, we used the date of the first outcome that occurred during the follow-up period. The median time to event and follow-up time, alongside the interquartile ranges, were reported for all outcomes. The Cox proportional hazard regression analyses produced estimated hazard ratios (HR) and corresponding 95% confidence intervals (CI). Schoenfeld residuals were plotted to verify whether the proportional hazards assumption was met. To evaluate the predictive accuracy of our models, we calculated and reported the concordance statistics (c-statistics), which gauge the area under the receiver operating characteristic curve. We simulated and visualized the HRs and 95% CI for late-life depression risk, for each age group, as a dose-response risk curve over the range of total BCS (0 to 20) employing a method by King, Tomz, and Wittenberg (18, 19). In this method, the mean BCS per group was considered the reference group, for which we executed 10,000 simulations per model.

#### Sensitivity analyses and secondary analyses

Sensitivity analyses involved *(A)* replicating the principal analysis with late-life depression being determined by READv2 and READv3 codes extracted from general practitioner data within a subset of the primary cohort, and *(B)* evaluating a potential bias due to the competing risks of death from other causes.

Secondary analyses included *(C)* statistically testing the variations in the associations of the BCS with the four outcomes across age and sex strata, *(D)* estimating the absolute risk across BCS quintiles. The secondary analyses *(C)* and *(D)* are presented in the supplementary information.

##### Sensitivity analyses: extension to a subpopulation with General-practitioner data

To evaluate the consistency of our results, we replicated the Cox proportional hazards analysis within a subset of the UK Biobank (UKB) cohort. This subset was specifically chosen as it uniquely contains data sourced from general practitioners (GPs), allowing for a different method of depression case ascertainment. Primary care data for approximately 230,000 UK Biobank participants (up until 2016 or 2017, contingent upon the data supplier) was released in 2019. This dataset comprises information from the GP system suppliers and includes coded clinical events (such as consultations, diagnoses, procedures, and laboratory tests), prescribed medications (including prescription date, drug code, and, when available, drug name and quantity), and a variety of administrative codes (for example, referrals to specialist hospital clinics). This data is coded using the READ2 and READ3 systems. Following a previously reported approach (20), we determined late-life depression cases based on the appearance of a list of codes listed in **Supplementary Information Table 14**.

##### Sensitivity analyses: competing risk of death due to other causes

In sensitivity analyses, we employed Fine and Gray subdistribution hazard models to estimate the association of the BCS with incident dementia, stroke, and the composite outcome, factoring in the competing risk of death due to any other cause (which prevents the occurrence of the outcome of interest). A substantial discrepancy between estimates from the main and sensitivity analyses would indicate a possible bias in the former (21). The Fine and Gray model analyses yielded subdistribution, cause-specific HRs, and 95% CI, with these HRs representing estimates of the relative difference in the rate of the event of interest’s (incident dementia, stroke, late-life depression or either of the three) occurrence among subjects who have not yet experienced the event of interest but may have experienced a competing event (18).

All statistical analyses were performed using R 4.2.1 (22). The current manuscript is written in line with the STROBE (Strengthening the Reporting of Observational Studies in Epidemiology) guidelines.

## Results

### Cohort characteristics

**Figure 2:**
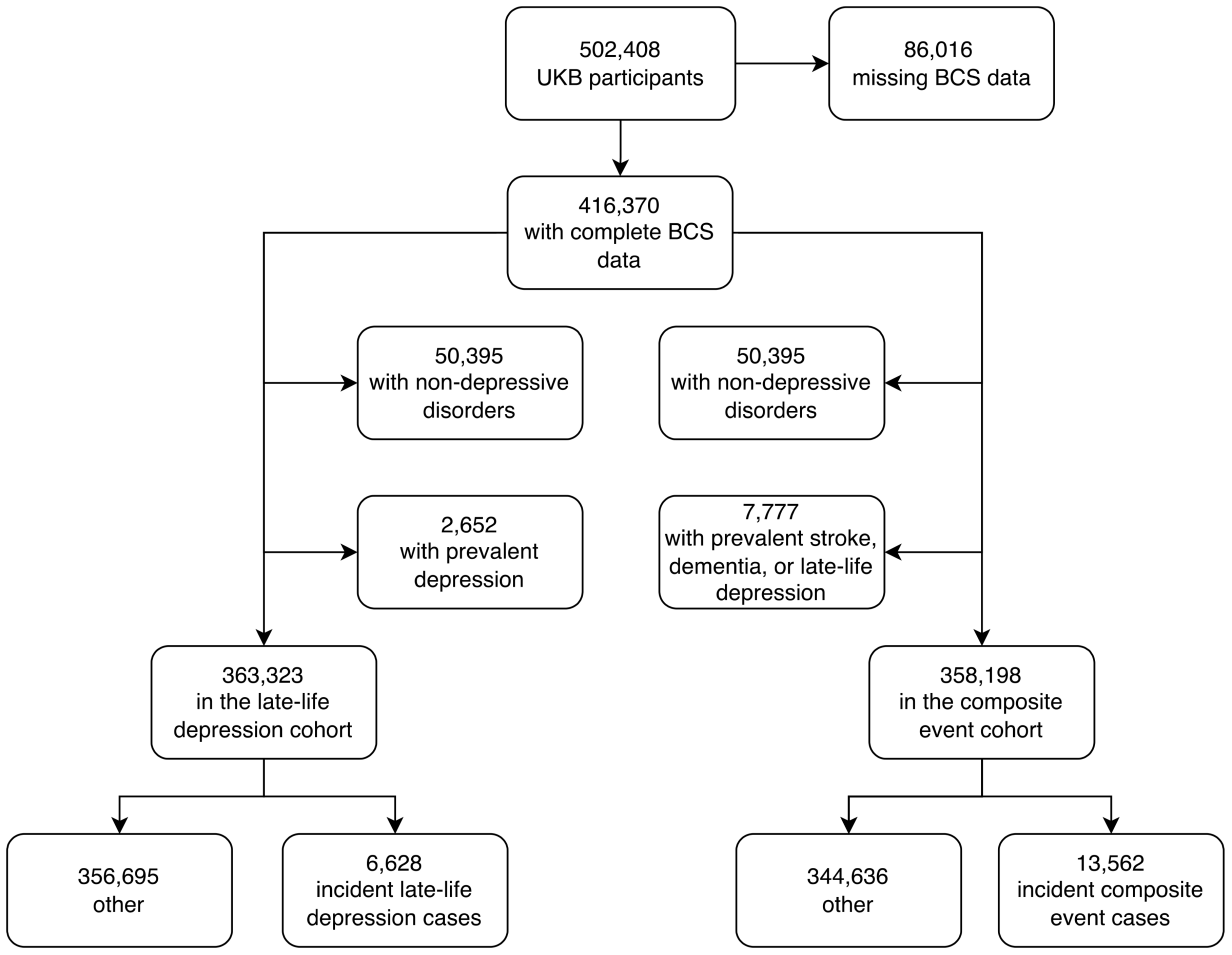
Flowchart for the late-life depression, and composite outcomes cohorts.

The UKB study has enrolled 502,408 participants between 2006 and 2010, for whom baseline measurements are available. We excluded 86,016 participants (17%) due to missing data on one or more of the individual BCS components. A total of 416,370 participants (mean age: 57, of which 54% were females) were included in the analyses (**Figure 2**).

To study late-life depression, we also excluded 50,395 UKB participants who had a mood or psychiatric disorder other than unipolar depression as well as 2,652 participants with depression at baseline or with a history of depression, and the final cohort for this analysis included 365,975 participants. When comparing the UKB participants included in the late-life depression study (who had complete data on the BCS and without a non-depressive mood or psychiatric disorder) with UKB participants excluded from the late-life depression study (who either were lacking data on the BCS or experienced a non-depressive mood or psychiatric disorder), there were fewer men in the study sample (45%) than in the excluded sample (48%), and the participants were younger in the study sample (56.4 years) than in the excluded sample (56.9 years) (**Supplementary Information Table S3**).

### Exposure: the BCS in the UK Biobank

**Figure 3:**
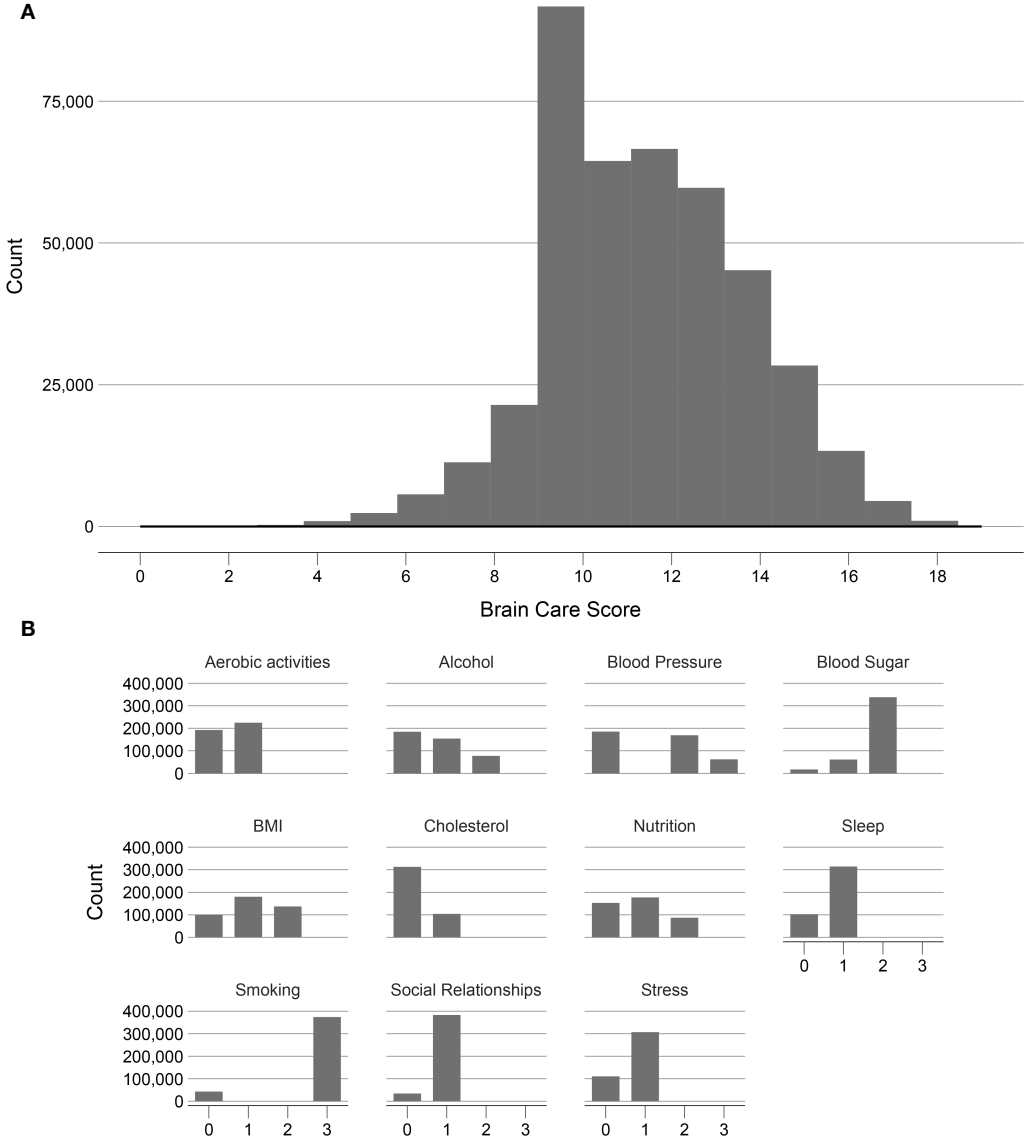
Frequency distribution of the Brain Care Score and its components in the UK Biobank. **(A)** shows the frequency of the total Brain Care Score over the range 1 to 19 observed in participants of the UKB study; **(B)** shows the frequencies of scores from the individual components of the total Brain Care Score.

The distributions of all UKB measurements making up the BCS are shown in **Figure 3**. Responses from the UKB questionnaires (mean and standard deviations, or frequencies for categorical variables), along with missingness percentage (%), stratified by three age categories (<50, 50-59, and >59 years), are provided in **Table 2**. The missingness for the BCS components at baseline ranged from 0.17% for physical activity to 7.2% for HbA1c.

**Table 2:** Cohort characteristics at baseline entry into the UKB.

	<50 years (n = 117,821)	50-59 years (n = 167,105)	>59 years (n = 217,460)	Overall (N = 502,386)
**Sex**				
Females	64,634 (54.9%)	93,998 (56.3%)	114,679 (52.7%)	273,311 (54.4%)
Males	53,187 (45.1%)	73,107 (43.7%)	102,781 (47.3%)	229,075 (45.6%)
**Age**				
Mean (SD)	45.0 (2.74)	54.8 (2.88)	64.1 (2.85)	56.5 (8.09)
**Systolic blood pressure**				
Mean (SD)	129 (15.9)	136 (17.7)	144 (18.7)	138 (18.7)
Missing	374 (0.3%)	408 (0.2%)	543 (0.2%)	1,325 (0.3%)
**Diastolic blood pressure**				
Mean (SD)	81.1 (10.4)	83.0 (10.2)	82.3 (10.0)	82.3 (10.2)
Missing	374 (0.3%)	408 (0.2%)	541 (0.2%)	1,323 (0.3%)
**Glycated haemoglobin (HbA1C) in %**				
Mean (SD)	5.27 (0.547)	5.45 (0.624)	5.56 (0.630)	5.46 (0.620)
Missing	8,713 (7.4%)	12,146 (7.3%)	15,133 (7.0%)	35,992 (7.2%)
**Cholesterol (mg/dL)**				
Mean (SD)	212 (39.4)	225 (43.0)	221 (47.1)	220 (44.3)
Missing	7,844 (6.7%)	10,966 (6.6%)	14,095 (6.5%)	32,905 (6.5%)
**BMI**				
Mean (SD)	27.0 (4.98)	27.5 (4.97)	27.6 (4.55)	27.4 (4.80)
Missing	763 (0.6%)	1,020 (0.6%)	1,321 (0.6%)	3,104 (0.6%)
**Fruit and vegetable servings per day**				
Mean (SD)	2.16 (4.08)	2.66 (3.85)	3.06 (3.59)	2.71 (3.81)
Missing	258 (0.2%)	287 (0.2%)	349 (0.2%)	894 (0.2%)
**Bread and cereal servings per day**				
Mean (SD)	2.17 (1.43)	2.22 (1.39)	2.40 (1.36)	2.28 (1.39)
Missing	1,286 (1.1%)	1,683 (1.0%)	1,697 (0.8%)	4,666 (0.9%)
**Red meat score**				
Mean (SD)	0.853 (0.989)	0.922 (1.03)	1.03 (1.06)	0.952 (1.03)
Missing	1,862 (1.6%)	2,131 (1.3%)	3,005 (1.4%)	6,998 (1.4%)
**Salt added to food**				
Always	6,524 (5.5%)	7,941 (4.8%)	9,959 (4.6%)	24,424 (4.9%)
Usually	12,577 (10.7%)	19,104 (11.4%)	26,699 (12.3%)	58,380 (11.6%)
Sometimes	33,456 (28.4%)	47,379 (28.4%)	59,753 (27.5%)	140,588 (28.0%)
Never/rarely	64,928 (55.1%)	92,338 (55.3%)	120,603 (55.5%)	277,869 (55.3%)
Missing	336 (0.3%)	343 (0.2%)	446 (0.2%)	1,125 (0.2%)
**Alcohol intake frequency**				
Daily or almost daily	17,721 (15.0%)	33,421 (20.0%)	50,608 (23.3%)	101,750 (20.3%)
Three or four times a week	27,274 (23.1%)	40,372 (24.2%)	47,771 (22.0%)	115,417 (23.0%)
Once or twice a week	33,953 (28.8%)	43,516 (26.0%)	51,793 (23.8%)	129,262 (25.7%)
One to three times a month	15,965 (13.6%)	18,412 (11.0%)	21,461 (9.9%)	55,838 (11.1%)
Special occasions only	13,205 (11.2%)	18,349 (11.0%)	26,439 (12.2%)	57,993 (11.5%)
Never	9,253 (7.9%)	12,548 (7.5%)	18,825 (8.7%)	406,26 (8.1%)
Missing	450 (0.4%)	487 (0.3%)	563 (0.3%)	1,500 (0.3%)
**Smoking status**				
Current	16,438 (14.0%)	18,623 (11.1%)	17,901 (8.2%)	52,962 (10.5%)
Previous	28,961 (24.6%)	53,882 (32.2%)	90,173 (41.5%)	173,016 (34.4%)
Never	71,800 (60.9%)	93,758 (56.1%)	107,902 (49.6%)	273,460 (54.4%)
Missing	622 (0.5%)	842 (0.5%)	1,484 (0.7%)	2,948 (0.6%)
**Days per week with 10+ minutes of moderate activity**				
Mean (SD)	3.25 (2.44)	3.26 (2.49)	3.53 (2.56)	3.37 (2.52)
Missing	255 (0.2%)	283 (0.2%)	340 (0.2%)	878 (0.2%)
**Days per week with 10+ minutes of vigorous activity**				
Mean (SD)	1.91 (2.02)	1.67 (2.02)	1.55 (2.05)	1.67 (2.04)
Missing	255 (0.2%)	283 (0.2%)	340 (0.2%)	878 (0.2%)
**Hours of sleep per day**				
Mean (SD)	7.07 (1.25)	7.01 (1.28)	7.19 (1.34)	7.10 (1.30)
Missing	255 (0.2%)	285 (0.2%)	347 (0.2%)	887 (0.2%)
**Number of days with tension, fidgetiness, or restlessness in the last two weeks**				
Nearly every day	3,062 (2.6%)	3,692 (2.2%)	2,585 (1.2%)	9,339 (1.9%)
Several days	29,995 (25.5%)	37,635 (22.5%)	37,798 (17.4%)	105,428 (21.0%)
More than half the days	4,184 (3.6%)	5,004 (3.0%)	4,238 (1.9%)	13,426 (2.7%)
Not at all	75,090 (63.7%)	113,721 (68.1%)	163,190 (75.0%)	352,001 (70.1%)
Missing	5,490 (4.7%)	7,053 (4.2%)	9,649 (4.4%)	22,192 (4.4%)
**Frequency of friends or family visits**				
Almost daily	9,991 (8.5%)	16,680 (10.0%)	31,085 (14.3%)	57,756 (11.5%)
2-4 times a week	30,288 (25.7%)	46,509 (27.8%)	75,205 (34.6%)	152,002 (30.3%)
About once a week	18,391 (15.6%)	24,557 (14.7%)	23,527 (10.8%)	66,475 (13.2%)
About once a month	46,002 (39.0%)	60,751 (36.4%)	69,617 (32.0%)	176,370 (35.1%)
Once every few months or never	11,032 (9.4%)	15,854 (9.5%)	14,968 (6.9%)	41,854 (8.3%)
Missing	2,117 (1.8%)	2,754 (1.6%)	3,058 (1.4%)	7,929 (1.6%)

Blood sugar levels were measured as haemoglobin A1c (HbA1C). The red meat score is based on beef, pork, and lamb/mutton consumption, in which an individual score was first assigned for each meat type (“Never” or “Less than once a week” with 0; “Once a week” or “2-4 times a week” with 1; and “5-6 times a week” or “Once or more daily” with 2); these were then summed, with a score of 1-2 dichotomized into 1 and less than 1 or more than 2 with a 0. Moderate activity includes physical activities such as carrying light loads or cycling; vigorous activity includes activities such as fast cycling, aerobics, or heavy lifting. BMI stands for Body Mass Index.

Of the included 365,975 UK participants, the median total BCS was 12 (total observed range: 2-19); with a median of 13 for participants aged <50 years, 12 for participants aged 50-59 years, and 12 for participants aged >59 years. There was a left-skewed distribution visible of the BCS (**Figure 3**).

### Risk of incident late-life depression

In total, after excluding 50,395 UKB participants who had a mood or psychiatric disorder other than depression and 2,652 prevalent cases of depression that occurred before baseline or in the first two years of follow-up, 6,628 incident cases of late-life depression were recorded (n = 363,323); the cumulative incidence of late-life depression was 1.8% (95% CI: 1.8-1.9), in line with previous findings (23, 24). The median time to event was 8.2 years and the median follow-up time was 12.5 years (**Supplementary Information Tables S4**, **5**). Older age was significantly associated with the incidence of late-life depression (p<0.001 when modelling age linearly). In stratified analyses, among participants aged <50 at baseline (n = 86,323), the cumulative incidence was 0.1% (95% CI: 0.1-0.1), with 81 late-life depression cases in total. The low incidence rate in this demographic is due to the ascertainment criteria for late-life depression that excludes cases in participants under the age of 60. Among those aged 50-59 years (n = 122,995), 2,313 cases of incident late-life depression were recorded, corresponding to a cumulative incidence of 1.9% (95% CI: 1.8-2.0). Among participants aged >59 (n = 154,005), the cumulative incidence was 2.7% (95% CI: 2.7-2.8), with 4,234 late-life depression cases.

### Association between the BCS and late-life depression

**Figure 4:**
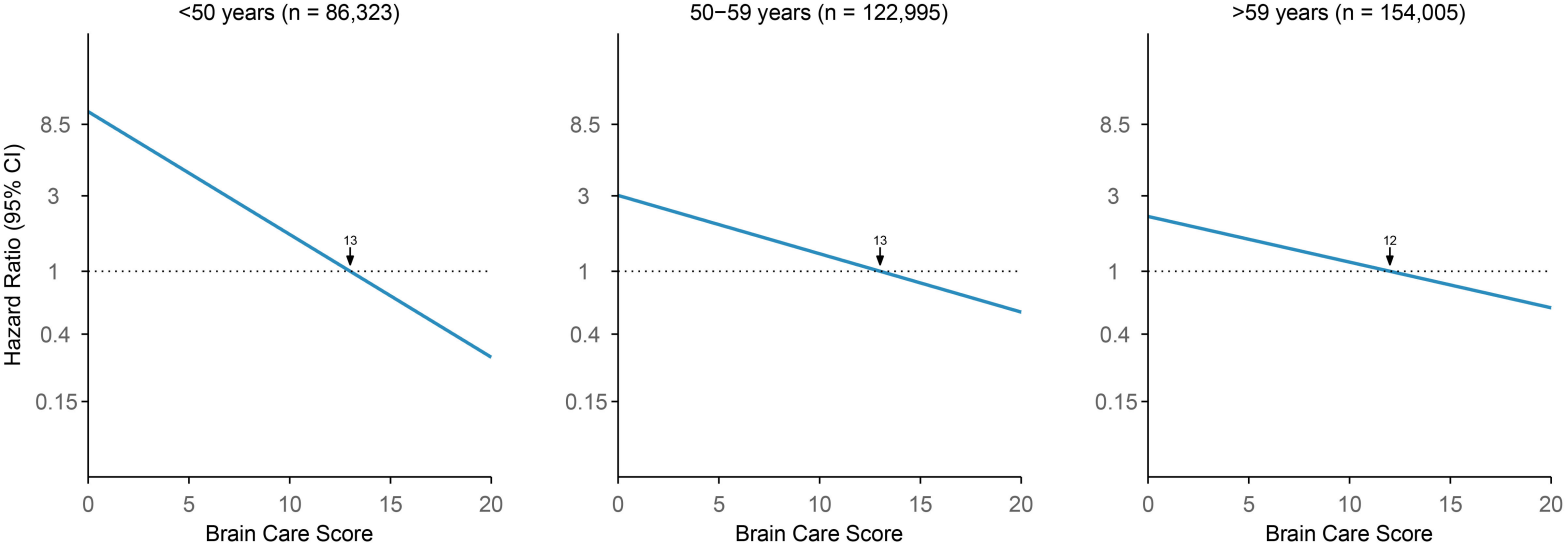
Association of Brain Care Score at baseline with incidence of late-life depression, stratified by age group at baseline. The thick line is the mean relative hazard curve for stroke incidence over the range of the Brain Care Score on a logarithmic scale; the shaded areas correspond to the 95% confidence intervals. The risk curves were adjusted for sex and plotted relative to the median Brain Care Score (indicated by the arrow) in the respective age group.

Each five-point increase in the baseline BCS was significantly associated with a 33% lower risk of incident late-life depression when adjusted for age and sex (HR: 0.67 [95% CI: 0.64-0.71], p-value: <0.01, c-statistic: 0.69; **Figure 4**; **Supplementary Information Table S6**). Age was a significant effect modifier of the association between the BCS and late-life depression (interaction p<0.001), with the BCS being associated with larger reductions in the risk of incident late-life depression among younger persons. Among participants aged <50 years at baseline, each five-point higher BCS was associated with a 59% lower risk of incident late-life depression (HR: 0.41, 95% CI: 0.26-0.63, p-value: <0.01, c-statistic: 0.63), adjusted for sex. Among those aged 50 to 59 years at baseline, each five-point increase in the BCS was associated with a 35% lower risk of late-life depression (HR: 0.65, 95% CI: 0.60-0.71, p-value: <0.01, c-statistic: 0.58), adjusted for sex. For participants aged >59 years at baseline, each five-point higher BCS was associated with a 28% lower risk of late-life depression (HR: 0.72, 95% CI: 0.67-0.77, p-value: <0.01, c-statistic: 0.58), adjusted for sex.

### Composite risk of incident late-life depression, dementia and stroke

Given the important role of dementia and stroke as other clinical endpoints related to brain health, we also tested for association between the BCS and the composite risk of late-life depression, dementia and stroke. Of note, the associations between the BCS and the risk of incident dementia and incident stroke have been previously reported (10). In total, after excluding 50,395 UKB participants who had a mood or psychiatric disorder other than depression and 7,777 prevalent cases of dementia *or* stroke *or* depression that occurred before baseline or in the first two years of follow-up, 13,562 incident cases of incident dementia *or* stroke *or* late-life depression were recorded (n = 358,198); the cumulative incidence of dementia or stroke or late-life depression was 3.8% (95% CI: 3.7-3.8). The median time to event was 8.7 years and the median follow-up time was 12.5 years (**Supplementary Information Tables S4**, **5**). Of the 13,562 cases, 798 occurred in participants who experienced at least two outcomes among stroke, dementia, or late-life depression. For these individuals, the date of the first diagnosis (dementia or stroke or late-life depression) was considered the time of event. Older age was significantly associated with the incidence of the combined outcome (p<0.001 when modelling age linearly). In stratified analyses, among participants aged <50 at baseline (n = 85,893), the cumulative incidence was 0.6% (95% CI: 0.6-0.7), with 518 stroke or dementia or late-life depression cases. Among those aged 50-59 years (n = 121,690), 3,589 cases of incident stroke or dementia or late-life depression were recorded corresponding to a cumulative incidence of 2.9% (95% CI: 2.9-3.0). Among participants aged >59 (n = 150,615), the cumulative incidence was 6.3% (95% CI: 6.2-6.4), with 9,455 incident stroke or incident dementia or incident late-life depression cases.

### Association between the BCS and the combined risk of incident stroke, dementia, and late-life depression

**Figure 5:**
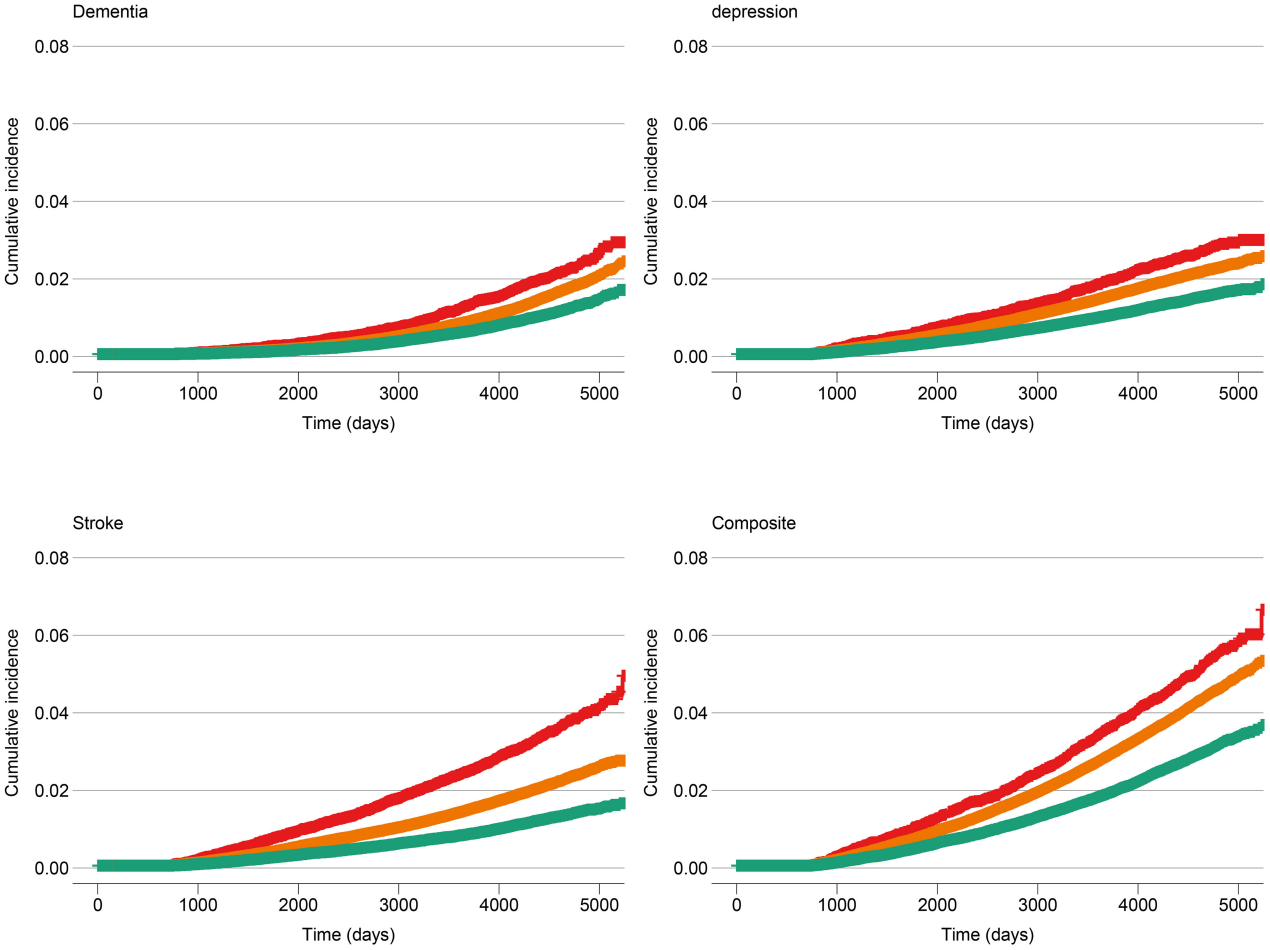
Cumulative incidence of dementia, stroke, late-life depression and dementia *or* stroke *or* late-life depression at baseline, stratified by Brain Care Score quintile group. The red line corresponds to the cumulative incidence of the low-scoring BCS group (1^st^ quintile: total BCS scores from 1 to 9), the orange line corresponds to the middle three BCS quintiles (total BCS scores from 10 to 13), and the green line corresponds to the high-scoring BCS group (5^th^ quantile: total BCS scores from 14 to 19).

Each five-point increase in the baseline BCS was associated with a 27% lower risk of incident stroke *or* incident dementia *or* late-life depression when adjusted for age and sex, and this difference was statistically significant (HR: 0.73 [95% CI: 0.70-0.76, p-value: <0.01, c-statistic: 0.71; **Supplementary Information Table S7**). Age was a significant effect modifier of the association between the BCS and the composite of late-life depression, dementia and stroke (interaction p<0.001), with the BCS being associated with larger reductions in the risk of incident stroke, dementia, or late-life depression among younger persons. Among participants aged <50 years at baseline, each five-point higher BCS was associated with a 38% lower risk of an event (HR: 0.62, 95% CI: 0.52-0.74, p-value: < 0.01, c-statistic: 0.59), adjusted for sex. Among those aged 50 to 59 years at baseline, each five-point increase in the BCS was associated with a 36% lower risk of an event (HR: 0.64, 95% CI: 0.60-0.69, p-value: <0.01, c-statistic: 0.56), adjusted for sex. Finally, for participants aged >59 years at baseline, each five-point higher BCS was associated with a 22% lower risk of an event (HR: 0.78, 95% CI: 0.75-0.82, p-value: <0.01, c-statistic: 0.53), adjusted for sex. Additionally, we demonstrated the cumulative incidence of dementia, stroke, or late-life depression stratified by BCS quintile groups (first quintile vs. second to fourth quintiles vs. fifth quintile) in **Figure 5**.

### Sensitivity analyses

#### Sensitivity analyses: extension to a subpopulation with general-practitioner data

Our primary analysis was replicated within a subgroup comprising 230,055 participants from the UK Biobank (UKB) for whom general-practitioner (GP) data were accessible. Of this subgroup, 192,468 participants had available BCS data, and 2,037 met the criteria for late-life depression based on READ3 codes (**Supplementary Information Figure S1**). Due to the timeframe of the general practitioner data collection, none of the participants who were 49 years or younger at enrolment in the UKB were given a depression code after turning 60, a prerequisite for defining late-life depression. The cumulative incidence of late-life depression within this subset was 1.1% (95% CI: 1.0-1.1%). The median time to event stood at 4.6 years, and the median follow-up duration was 12.4 years. Among those aged 50-59 years (n = 64,615), there were 597 incident cases of late-life depression, corresponding to a cumulative incidence of 0.9% (95% CI: 0.9-1.0). In participants aged over 59 years (n = 80,974), the cumulative incidence was 1.8% (95% CI: 1.7-1.8), with 1,440 recorded cases of late-life depression.

Each five-point increase in the baseline BCS was associated with a 40% lower risk of incident late-life depression when adjusted for age and sex, and this difference was statistically significant (HR: 0.60 [95% CI: 0.50-0.69], p-value: <0.01, c-statistic: 0.72; **Supplementary Information Figure S2**, **Supplementary Information Table S11**). Among those aged 50 to 59 years at baseline, each five-point increase in the BCS was associated with a 41% lower risk of late-life depression (HR: 0.59, 95% CI: 0.43-0.76, p-value: <0.01, c-statistic: 0.57), adjusted for sex. For participants aged >59 years at baseline, each five-point higher BCS was associated with a 38% lower risk of late-life depression (HR: 0.62, 95% CI: 0.50-0.73, p-value: <0.01, c-statistic: 0.56), adjusted for sex.

#### Sensitivity analyses: competing risk of death due to other causes and proportional hazards assumption

In sensitivity analyses, Fine and Gray subdistribution hazard models (21) were used to assess the effect of competing risk of death due to other causes. The subdistribution (cause-specific) HR estimates did not differ substantially from estimates from the main Cox regression analyses (**Supplementary Information Tables S12**, **S13**). Schoenfeld residuals were plotted in **Supplementary Information Figures S3**, **S4**: no pattern with time is visible for HR estimates, although some of the associated p-values indicated statistical significance, which was unsurprising given the sample sizes.

## Discussion

Herewith, we present clinically relevant and statistically significant associations between the BCS, a novel tool to promote brain care, and late-life depression using hospital-based data and GP data from the UKB cohort. In addition, the BCS strongly associates with a composite trait of major brain health outcomes: stroke, dementia and late-life depression. The associations between BCS and late-life depression, as well as the brain health composite trait, were consistent across all included age groups. Furthermore, these associations were verified with a different ascertainment of late-life depression using data from general practitioners within a subset of the UKB cohort.

The strengths and weaknesses of the BCS as a novel tool to be implemented into routine primary care have been described before (10). For the currently presented analyses, multiple strengths include our well-powered sample, outcome ascertainment for late-life depression in both hospital-based and GP datasets, and the close approximation of BCS factors found in the UKB. However, several limitations of the current analyses should be addressed as well. First, there is an ongoing debate on the causality and directionality between some of the individual components of the BCS with late-life depression (e.g., the social-emotional components). One of the drawbacks is that the mechanisms as to which social-emotional stressors lead to vascular disease or neurodegeneration remain largely unknown, although hypotheses on the roles of amygdalar activity (25–27) and/or neurobiological resistance (28) have been published. There may be risk factors for late-life depression that are not risk factors for dementia and stroke (and therefore not included in the BCS) such as abuse or trauma during childhood (29). However, no consensus has been achieved on the role of all psychiatric orders being risk factors for dementia. For example, recent compelling data has been published on the relationship between post-traumatic stress syndrome (PTSD), psychotic disorders and bipolar disorder (BPD) with onset of dementia (30–32). Furthermore, we acknowledge the existence of several diagnostic instruments specifically designed for the detection of depression in geriatric populations. These include the Beck’s depression inventory-II (BDI-II) (33), the Center for Epidemiologic Studies Depression Scale (CES-D) (34), the Hamilton depression scale (HAM-D) (35) and the geriatric depression scale (GDS) (36). The BCS, however, was primarily developed to incorporate brain care in routine primary practice, rather than serving as a diagnostic tool. In addition, to our knowledge, there is currently no clinical tool to predict late-life depression, and the concordance statistics we obtained suggest that the BCS has strong predictive abilities. Consequently, we refrain from drawing direct comparisons between the BCS and the aforementioned depression scales – as the aims and implementation of these scores differ significantly.

Regarding outcome ascertainment within the hospital-based and GP-based UK Biobank (UKB) cohort, the application of a well-validated ICD-code-based ascertainment of late-life depression (15, 16) yielded highly consistent results with an ascertainment derived from a GP-based dataset. It is worth noting that GPs frequently act as the first point of contact for diagnosing depression (37, 38), with a substantial proportion of patients primarily seeking their GPs’ assistance in managing depressive symptoms, rather than consulting psychiatric specialists. This replication strategy strengthens our findings by ensuring they apply not only to those diagnosed in a hospital setting but also to a broader population of individuals who primarily interact with the general healthcare system.

Importantly, emerging evidence suggests that depression is a risk factor for cognitive decline and dementia (39–42). A recent review proposed the following biological explanation for this: depression activates pro-inflammatory mediators, leading to cerebral small vessel disease (SVD) with reduced cerebral blood flow: the latter being a well-studied precedent of cognitive decline and dementia (41). The incidence of depression is rising, with adolescents reported to be at the highest risk of developing a depression, and with the onset of depression occurring at increasingly earlier ages (41, 43–45). The US department of Health and Human Services reported in 2020 that the age groups suffering most from depression symptoms were people aged 18–29 (21%), followed by 45–64 years and >65 years (18%), and 30–44 years (17%). Women are more likely to be diagnosed with depression compared to men across all age categories (46). Although no global consensus has been established on this, early-life depression has oftentimes been proposed as a risk factor for dementia (47–49), and late-life depression as a prodrome of dementia (40, 47, 48). Prevention and adequate treatment of early-life (and potentially late-life) depression may not only be effective in reducing people’s suffering directly, but could also indirectly lower the incidence of cognitive decline and dementia (40). Additional research is needed to establish the causality between depression prevention/treatment and lower dementia incidence.

Hence, implementation of the BCS into routine primary care, and thus stimulating people from all ages to take better care of their brains (subsequently leading to health behaviours that reduce the risk of dementia, stroke and depression) could be an effective way to holistically improve quality of life for middle-aged individuals. There may be a wide range of positive consequences of implementing the use of the BCS in primary prevention worldwide. Importantly, it could educate both patients and practitioners on the preventability and modifiable risk factors for dementia, stroke, and depression. Ultimately, this could lead to a “snowballing effect” of the BCS as a simple presentation of the modifiable risk factors for these brain diseases to the general population. In the future, we aim to validate the BCS in different settings (languages, cultures, geographical locations etc). The definitions (e.g., dietary component) of some of the individual components may need to be adjusted based on a specific different setting, as well as the weighing (e.g., different effects of BMI for different ethnicities).

To conclude, we present the first-ever analyses between the BCS and incident late-life depression and a brain health composite trait in 358,198 individuals from the UKB – in line with previously published data on the BCS with dementia and stroke incidence in UKB. Continuous revision and optimisation of the BCS needs to be routinely performed via a cycle, based on ongoing research on individual BCS components and its associations with dementia, stroke, and late-life depression (leading to different weighing of individual components, as well as adding or removing components as the scientific evidence grows), as well as expert consensus (e.g., via the Delphi process). Importantly, research on the motivational aspects of the BCS for different age groups and populations is warranted if we are to be successful in achieving sustainable behaviour changes towards risk factor reduction of brain diseases. The primary and secondary prevention of dementia, stroke and depression through risk factor modification and behaviour change is fundamental to improve populations’ brain health, reduce health inequalities, and contain healthcare costs worldwide (13). The BCS is a prototype tool to achieve these goals via implementation into routine primary care.

## Data Availability

Publicly available datasets were analysed in this study. This data can be found here: https://www.ukbiobank.ac.uk. This analysis was approved by the UK Biobank access committee as part of project 58743.
