# Corrigendum: The predictive validity of a Brain Care Score for late-life depression and a composite outcome of dementia, stroke, and late-life depression: data from the UK Biobank cohort

**DOI:** 10.3389/fpsyt.2024.1502482

**Published:** 2024-11-12

**Authors:** Sanjula D. Singh, Cyprien A. Rivier, Keren Papier, Zeina Chemali, Leidys Gutierrez-Martinez, Livia Parodi, Ernst Mayerhofer, Jasper Senff, Santiago Clocchiatti-Tuozzo, Courtney Nunley, Amy Newhouse, An Ouyang, M. Brandon Westover, Rudolph E. Tanzi, Ronald M. Lazar, Aleksandra Pikula, Sarah Ibrahim, H. Bart Brouwers, Virginia J. Howard, George Howard, Nirupama Yechoor, Thomas Littlejohns, Kevin N. Sheth, Jonathan Rosand, Gregory Fricchione, Christopher D. Anderson, Guido J. Falcone

**Affiliations:** ^1^ Henry and Allison McCance Center for Brain Health, Massachusetts General Hospital, Boston, MA, United States; ^2^ Department of Neurology, Massachusetts General Hospital, Boston, MA, United States; ^3^ Broad Institute of Massachusetts Institute of Technology (MIT) and Harvard, Cambridge, MA, United States; ^4^ Department of Neurology, Yale School of Medicine, New Haven, CT, United States; ^5^ Yale Center for Brain and Mind Health, New Haven, CT, United States; ^6^ Nuffield Department of Population Health, University of Oxford, Oxford, United Kingdom; ^7^ Division of Neuropsychiatry, Massachusetts General Hospital, Boston, MA, United States; ^8^ Center for Genomic Medicine, Massachusetts General Hospital, Boston, MA, United States; ^9^ Department of Neurology, Brigham and Women’s Hospitall, Boston, MA, United States; ^10^ Department of Neurology, Rudolf Magnus Institute of Neuroscience, University Medical Centre Utrecht, Utrecht, Netherlands; ^11^ Department of Medicine, Massachusetts General Hospital, Boston, MA, United States; ^12^ Department of Neurology, University of Alabama at Birmingham (UAB) Heersink School of Medicine, University of Alabama at Birmingham (UAB) McKnight Brain Institute, Birmingham, AL, United States; ^13^ Jay and Sari Sonshine Centre for Stroke Prevention & Cerebrovascular Brain Health, University Health Network, Krembil Brain Institute, Toronto, ON, Canada; ^14^ Department of Medicine, Division of Neurology, The Temerty Faculty of Medicine at the University of Toronto, Toronto, ON, Canada; ^15^ Program for Health System and Technology Evaluation; Toronto General Hospital Research Institute, Toronto, ON, Canada; ^16^ Institute of Health Policy, Management and Evaluation (IHPME), Dalla Lana School of Public Health; University of Toronto, Toronto, ON, Canada; ^17^ Centre for Advancing Collaborative Healthcare & Education (CACHE), University of Toronto, Toronto, ON, Canada; ^18^ Department of Neurosurgery, Elisabeth TweeSteden Ziekenhuis, Tilburg, Netherlands; ^19^ Department of Biostatistics, School of Public Health, University of Alabama at Birmingham, Birmingham, AL, United States; ^20^ Benson-Henry Institute for Mind Body Medicine, Massachusetts General Hospital, Boston, MA, United States

**Keywords:** depression - epidemiology, prevention, risk factor, brain health, stroke, dementia

In the published article there was a error in the **Conflict of interest** statement for M. Brandon Westover. The statement was previously:

“CA receives sponsored research support from the US National Institutes of Health, the American Heart Association, and Bayer AG, and has consulted for ApoPharma. GF receives sponsored research support from the National Institute of Mental Health Clinical Global Mental Health Research T32 Fellowship, receives royalties or licenses from Johns Hopkins University Press, University of Chicago Press, Belvoir Press, and the American Psychiatric Press, is on a Data Safety Monitoring Board or Advisory Board of Healthy Hearts Healthy Minds DSMB, is a Board of Directors member at the Rosalynn Carter Institute, and has stock or stock options from Revival Therapeutics Consultant. LG-M receives sponsored research support from the American Heart grant number 963719. JR receives sponsored research support from the US National Institutes of Health and the American Heart Association, and receives payments for expert testimony and consulting fees from the National Football League. EM is an employee of Regeneron Genetics Center.

The remaining authors declare that the research was conducted in the absence of any commercial or financial relationships that could be construed as a potential conflict of interest.​

The author(s) declared that they were an editorial board member of Frontiers, at the time of submission. This had no impact on the peer review process and the final decision.”

The correct statement appears below:

“CA receives sponsored research support from the US National Institutes of Health, the American Heart Association, and Bayer AG, and has consulted for ApoPharma. GF receives sponsored research support from the National Institute of Mental Health Clinical Global Mental Health Research T32 Fellowship, receives royalties or licenses from Johns Hopkins University Press, University of Chicago Press, Belvoir Press, and the American Psychiatric Press, is on a Data Safety Monitoring Board or Advisory Board of Healthy Hearts Healthy Minds DSMB, is a Board of Directors member at the Rosalynn Carter Institute, and has stock or stock options from Revival Therapeutics Consultant. LG-M receives sponsored research support from the American Heart grant number 963719. JR receives sponsored research support from the US National Institutes of Health and the American Heart Association, and receives payments for expert testimony and consulting fees from the National Football League. EM is an employee of Regeneron Genetics Center. MW has private equity as co-founder of Beacon Biosignals and receives compensation for consulting and scientific advisory roles.

The remaining authors declare that the research was conducted in the absence of any commercial or financial relationships that could be construed as a potential conflict of interest.​

The author(s) declared that they were an editorial board member of Frontiers, at the time of submission. This had no impact on the peer review process and the final decision.”

The authors apologize for this error and state that this does not change the scientific conclusions of the article in any way. The original article has been updated.

