# Supplementary material for: The predictive validity of a Brain Care Score for late-life depression and a composite outcome of dementia, stroke, and late-life depression: data from the UK Biobank cohort
Source: Front Psychiatry. 2024 Jul 23;15:1373797. doi: 10.3389/fpsyt.2024.1373797 (PMC11301016; doi:10.3389/fpsyt.2024.1373797)
Supplement: Supplementary file 1 [file DataSheet1.pdf]

Supplementary Information for "A Brain Care Score for Risk of Late-Life Depression: data from the UK Biobank cohort"

This appendix provides supplementary methods and results for "A Brain Care Score for Risk of Late-Life Depression: data from the UK Biobank cohort".

Table of Content

Supplementary methods 2

Supplementary results 3

Supplementary Tables 4

Table S1. Differences between the BCS and UKB derived BCS 4

Table S2. Sample characteristics at baseline among participants with and without complete Brain Care Score data 6

Table S3. Sample characteristics at baseline among participants included in the late-life depression study and excluded from it 6

Table S4. Median follow-up time in years for late-life depression, and composite outcome analyses 7

Table S5. Median time to event in years for late-life depression, and composite outcome analyses 7

Table S6. Coefficient table for late-life depression models 7

Table S7. Coefficient table for composite models 8

Table S8. Hazard Ratios (95%CI) between the Brain Care Score and risk of late-life depression and dementia *or* stroke *or* late-life depression 9

Table S9. Brain Care Score components, by quintile group 10

Table S10. Cumulative incidence of late-life depression, and dementia or stroke or late-life depression at baseline by Brain Care Score quintile group 12

Table S11. Coefficient table for late-life depression models in the GP data 13

Table S12. Coefficient table for late-life depression Fine and Gray subdistribution hazard model 13

Table S13. Coefficient table for composite event Fine and Gray subdistribution hazard model 13

Table S14. READ2 and READ3 codes used to ascertain depression in the General Practitioner subset 14

Supplementary Figures 15

Fig. S1 | Flowchart for the General Practitioner data cohort 15

Fig. S2 | Cumulative incidence of late-life depression in the GP data cohort, stratified by Brain Care Score quintile group 16

Fig. S3 | Schoenfeld residuals for proportional hazards assumption with incident late-life depression as outcome 17

Fig. S4 | Schoenfeld residuals for proportional hazards assumption with incident dementia *or* stroke *or* late-life depression as outcome 19

Bibliography 21

Supplementary methods

Outcome assessment: incident dementia and incident stroke

Utilizing the consistently collected hospital and mortality data, the UK Biobank Outcome Adjudication Group, in conjunction with clinical experts, established algorithms to identify instances of all-cause dementia and stroke. The comprehensive list of codes from the International Classification of Disease, 10th and 9th editions (ICD-10, ICD-9), utilized in the algorithmic definition of dementia and stroke cases, can be accessed on the UK Biobank website.

Secondary analyses

C. Testing the differences in the associations of the BCS across groups (age groups and sex)

Using Cox proportional hazard models that integrated two- and three-way interactions among the BCS, age, and sex, we tested the variations in BCS associations across groups, as documented in the main analyses.

D. Absolute risk estimates across quintiles of the BCS

Absolute risk estimates and variations in absolute risk across BCS ranges were provided, in addition to relative risk estimates derived from Cox proportional hazard models. The cumulative incidence and 95% confidence interval (CI) in participants with a "low BCS" indicative of suboptimal brain care (defined as 1st quintile), with a "medium BCS" (defined as 2nd, 3rd, and 4th quintiles), and participants with a "high BCS" suggesting optimal brain care (defined as 5th quintile) were reported. The Agresti-Coull method[1] was utilized to estimate the 95% CI of the cumulative incidence of dementia, stroke, and late-life depression for all participants as well as those stratified by age categories.

Supplementary results

Comparison between participants with complete and incomplete data

When comparing the UKB participants who had complete data on the BCS (*included in this study*) with UKB participants without complete data on the BCS (*excluded from this study*), there were more men in the study sample (46%) than in the excluded sample (44%), but no substantial differences in age (Supplementary Information Table S2).

Secondary analyses

C. *Testing the differences in the associations of the BCS across groups (age and sex)*

In secondary analyses statistically testing any differences in associations across the three age groups and sex, two-way interactions between the BCS and age were directionally consistent with the age-stratified primary analyses and statistically significant for some age groups. The interactions of the BCS with sex were close to the null hypothesis (HR of 1) and highly uncertain, as were three-way interactions (Supplementary Information Table S8).

D. *Absolute risk estimates across quintiles of the BCS*

In a secondary analysis, there was a substantial decrease in absolute risk of the four outcomes (incident dementia, incident stroke, incident late-life depression and incident dementia or stroke or late-life depression) for participants with a higher total baseline BCS. Individuals were grouped into three categories for analysis: a low-scoring group, with a BCS in the 1st quintile (total BCS scores of 1 to 9); a group with a BCS in the middle three quintiles (with total BCS scores of 10 to 13); and a high-scoring group, with a BCS in the 5th quintile (14 to 19), the highest scores observed in the sample (see Supplementary Information Table S9 for BCS components by quintile groups). The cumulative incidence of all four outcomes was lower in the high-scoring group than in the middle- and – even more substantially – in the low-scoring groups (see Fig. 5 and Supplementary Information Table S10).

Supplementary Tables

Table S1. Differences between the BCS and UKB derived BCS

Measurement	Information
**Blood Pressure**	
Differences BCS and UKB-BCS	None
UKB data to score conversion	The mean of two automated, or, if not available, manual blood pressure measurements was calculated and used to score blood pressure
**Blood Glucose**	
Differences BCS and UKB-BCS	None
UKB data to score conversion	UKB data in mmol/mol was converted to % using the formula: AIC(%) = (A1C(mmol/mol)/10.929)+2.15
**Cholesterol**	
Differences BCS and UKB-BCS	None
UKB data to score conversion	UKB data in mmol/L was converted to mg/dL using the formula: mmol/L*38.66976
**Body Mass Index**	
Differences BCS and UKB-BCS	None
UKB data to score conversion	None
**Nutrition**	
Differences BCS and UKB-BCS	• 2 servings of lean protein per day was translated into 1–4 servings of red meat per week<br>• 3 or more servings of whole grains per day was translated into 3 or more servings of bread slices or cereal bowls per day<br>• Less than 1500mg of sodium per day was translated into sometimes, rarely or never added salt to a meal<br>• Less than 36 0z. of sugar sweet beverages (soda, juice, etc.) per week was not included as it was not collected in the baseline assessment of the UKB<br>• Alternation was necessary to avoid significant power loss<br>• Self-reports were used
UKB data to score conversion vegetables and fruit	• To convert the reported intake of cooked and salad/raw vegetables from heaped tablespoons to standard serving sizes, we used the American Heart Association's (AHA) Serving Sizes guidelines[42]<br>• UKB-data heaped tablespoons were translated into 2 tablespoons<br>• 8 heaped tablespoons or 16 tablespoons are equivalent to 1 cup<br>• 1 serving of cooked vegetables consists of 4 heated tablespoons equivalent to ½ cup<br>• 1 serving of salad/raw vegetables consists of 8 heaped tablespoons equivalent to 1 cup<br>• 1 serving of fruit consisted of 2 dried fruit portions<br>• At least 4.5 half servings, as stated above, were considered a dietary recommendation[23]
UKB data to score conversion red meat	• Beef, pork, and lamb/mutton consumption was combined to a red meat score, in which an individual score was first assigned for each meat type and later combined to a total number of red meat servings per week<br>• "Never" or "less than once a week" for each type of red meat were calculated as 0 points<br>• "Once a week" or "2–4 times per week" for each type of red meat were calculated as 1 point<br>• "5–6 times a week" or "Once or more daily" for each type of red meat were calculated as 2 points<br>• A total score of 1–2 points was considered a dietary recommendation, a score of 0 or more than 2 was not
**Alcohol consumption**	
Differences BCS and UKB-BCS	• 4 or more alcoholic drinks per week was translated into 3 or more times of alcohol consumption per week<br>• 2–3 alcoholic drinks per week was translated into 1–2 times of alcohol consumption per week or 1–3 times of alcohol consumption per month<br>• 0–1 alcoholic drink per week was translated into never/ special occasions only
UKB data to score conversion	• 3 or more times of alcohol consumption per week is 0 points<br>• 1–2 times per week or 1–3 times per month is 1 point<br>• "Never" or "only on special occasions" is 2 points

	• Alternation was necessary to avoid significant power loss<br>• Self-reports were used
**Smoking**	
Differences BCS and UKB-BCS	Never smoked or quit more than a year ago was translated into non-smoker
UKB data to score conversion	• Non-smokers are a combination of former smokers and never having smoked<br>• Alternation was necessary, given the lack of data on the time of stopped smoking, to avoid significant power loss<br>• Self-reports were used
**Aerobic activities**	
Differences BCS and UKB-BCS	• Less than 150 minutes of moderate or 75 minutes of high intensity physical activity per week was translated into at least 10 minutes of moderate or vigorous activity for less than 5 days per week<br>• At least 150 minutes of moderate physical activity (ex. walking) or 75 minutes of high intensity physical activity per week was translated into at least 10 minutes of moderate or vigorous activity for at least 5 days per week
UKB data to score conversion	• At least 10 minutes of moderate or vigorous activity for less than 5 days per week is 0 points<br>• At least 10 minutes of moderate of vigorous activity for at least 5 days per week is 1 point<br>• Moderate activity includes physical activities such as carrying light loads or cycling; vigorous activity includes activities such as fast cycling, aerobics, or heavy lifting<br>• Self-reports were used
**Sleep**	
Differences BCS and UKB-BCS	• Untreated sleep disorder and/or sleep <7 hours per night was translated into at less than 7 hours of sleep per day<br>• Treated sleep disturbances and 7–8 hours of routine sleep per night was translated into at least 7 hours of sleep per day
UKB data to score conversion	• Less than 7 hours per day of sleep is 0 points<br>• At least 7 hours per day of sleep is 1 point<br>• Data on sleep disorders (treated or untreated) was not available at baseline measurement of the UKB<br>• Self-reports were used
**Stress**	
Differences BCS and UKB-BCS	• Moderate level of stress that occasionally makes it difficult to function and high level of stress that often makes it difficult to function were both translated into the presence of self-perceived tension, fidgetiness or restlessness in the last 2 weeks<br>• Manageable level of stress that rarely makes it difficult to function was translated into the absence of self-perceived tension, fidgetiness or restlessness in the last 2 weeks
UKB data to score conversion	• The presence of self-perceived tension, fidgetiness or restlessness in the last two weeks is 0 points<br>• The absence of self-perceived tension, fidgetiness or restlessness in the last two weeks is 1 point<br>• As the definition used in the UKB-BCS did not completely match the BCS we scored 0–1, instead of 0–2<br>• Self-reports were used
**Social relationship**	
Differences BCS and UKB-BCS	• I have few or no close connections other than my spouse or children was translated into visits from family or friends outside household less than once a month<br>• I have at least two people, other than my spouse or children, that I feel close with and could talk about private matters or call upon for help was translated into visits from family or friends outside household at least once a month
UKB data to score conversion	• The social relationship component was captured by the frequency of friends or family visits<br>• No friends or family members outside the household, no or almost no visits, or only once every few months is a score of 0 points<br>• Visits once a month, once a week, two to four times a week, or almost daily are a score of 1 point<br>• Self-reports were used

**Meaning in life**	Due to a lack of information on the self-reported meaning of life in the baseline measurements of the UKB study, we were unable to quantify a corresponding factor and therefore did not include this component

Legend. BCS = original Brain Care Score, UKB = United Kingdom Biobank, UKB-BCS = United Kingdom Biobank derived Brain Care Score, HbA1c = haemoglobin A1c.

Table S2. Sample characteristics at baseline among participants with and without complete Brain Care Score data

	Complete BCS data	Incomplete BCS data
	% (95% CI)	
Males	0.46 (0.46 to 0.46)	0.44 (0.44 to 0.45)
	Mean (95% CI)	
Age	56.45 (56.42 to 56.47)	56.85 (56.8 to 56.9)

Legend. 95% CI = 95% confidence interval.

Table S3. Sample characteristics at baseline among participants included in the late-life depression study and excluded from it

	Included in the study	Excluded from the study
	% (95% CI)	
Males	0.45 (0.44 - 0.45)	0.48 (0.48 - 0.48)
	Mean (95% CI)	
Age	56.39 (56.36 - 56.41)	56.9 (56.86 - 56.95)

Table S4. Median follow-up time in years for late-life depression, and composite outcome analyses

	Median follow-up time for late-life depression analysis (IQR)	Median follow-up time for the composite outcome (IQR)
**Overall**	12.54 (11.8 - 13.23)	12.53 (11.8 - 13.22)
**Age < 50**	12.61 (11.9 - 13.28)	12.6 (11.9 - 13.27)
**Age 50-59**	12.59 (11.86 - 13.27)	12.58 (11.85 - 13.25)
**Age > 59**	12.43 (11.63 - 13.16)	12.41 (11.63 - 13.14)

Legend. IQR = interquartile range.

Table S5. Median time to event in years for late-life depression, and composite outcome analyses

	Median follow-up time to late-life depression diagnosis (IQR)	Median follow-up time to event for the composite outcome (IQR)
**Overall**	8.24 (5.57 - 10.46)	8.72 (6.02 - 10.83)
**Age < 50**	12.35 (11.51 - 12.95)	9.38 (6.14 - 11.38)
**Age 50-59**	8.77 (6.39 - 10.65)	8.76 (6.27 - 10.76)
**Age > 59**	7.87 (5.18 - 10.23)	8.68 (5.91 - 10.81)

Legend. IQR = interquartile range.

Table S6. Coefficient table for late-life depression models

	**Incident late-life depression**			
	**Overall**	**<50 years**	**50-59 years**	**>59 years**
Per 5-point increase in Brain Care Score	0.67***	0.41***	0.65***	0.72***
	(0.64 to 0.71)	(0.26 to 0.63)	(0.60 to 0.71)	(0.67 to 0.77)
Age	1.09***			
	(1.09 to 1.10)			
Sex: males	0.61***	0.52**	0.63***	0.61***
	(0.58 to 0.64)	(0.32 to 0.83)	(0.58 to 0.69)	(0.57 to 0.65)
Observations	363,323	86,323	122,995	154,005
Cases	6,628	81	2,313	4,234

Legend. Reference group: females (sex).
*p<0.05; **p<0.01; ***p<0.001

Table S7. Coefficient table for composite models

	Incident dementia *or* stroke *or* late-life depression			
	**Overall**	**<50 years**	**50-59 years**	**>59 years**
Per 5-point increase in Brain Care Score	0.73***	0.62***	0.64***	0.78***
	(0.70 to 0.76)	(0.52 to 0.74)	(0.60 to 0.69)	(0.75 to 0.82)
Age	1.11***			
	(1.11 to 1.11)			
Sex: Males	0.95*	1.39***	0.90**	0.98
	(0.92 to 0.99)	(1.16 to 1.66)	(0.85 to 0.97)	(0.94 to 1.02)
Observations	358,198	85,893	121,690	150,615
Cases	13,562	518	3,589	9,455

Legend. Reference group: females (sex).
*p<0.05; **p<0.01; ***p<0.001

Table S8. Hazard Ratios (95%CI) between the Brain Care Score and risk of late-life depression and dementia *or* stroke *or* late-life depression

	Incident late-life depression	Incident dementia *or* stroke *or* late-life depression
Per 5-point increase in Brain Care Score	0.35***	0.54***
	(-0.19 to 0.88)	(0.28 to 0.15)
Age group: >59 years	4.71**	5.74***
	(3.35 to 6.06)	(5.02 to 6.45)
Age group: 50-59 years	3.78*	3.82***
	(2.41 to 5.15)	(3.09 to 4.56)
Sex: males	0.19	0.80
	(-2.09 to 2.47)	(-0.11 to 1.72)
BCS x Age group: >59 years	2.06***	1.38**
	(1.52 to 2.60)	(1.10 to 1.65)
BCS x Age group: 50-59 years	1.88**	1.18
	(1.34 to 2.43)	(0.90 to 1.47)
BCS x Sex: males	1.54	1.25
	(0.60 to 2.48)	(0.89 to 1.61)
Age group: >59 years x Sex: males	3.18	0.96
	(0.88 to 5.49)	(0.02 to 1.90)
Age group: 50-59 years x Sex: males	3.34	1.13
	(1.02 to 5.67)	(0.15 to 2.11)
BCS x Age group: >59 years x Sex: males	0.66	0.89
	(-0.29 to 1.60)	(0.51 to 1.26)
BCS x Age group: 50-59 years x Sex: males	0.65	0.80
	(-0.30 to 1.61)	(0.41 to 1.19)
Observations	363,323	358,198
Cases	6,628	13,562

The table shows the Hazard Ratios and corresponding 95% Confidence Intervals of associations between the Brain Care Score and risk of dementia, stroke, and dementia *or* stroke featuring two- and three-way interactions between the BCS, age groups, and sex.

Table S9. Brain Care Score components, by quintile group

	1st quintile (n = 79,690)	2nd-4th quintile (n = 288,479)	5th quintile (n = 47,201)	Overall (N = 416,370)
**Sex**				
Female	34890 (43.8%)	159197 (55.0%)	31233 (66.2%)	225320 (54.1%)
Male	44800 (56.2%)	130282 (45.0%)	15968 (33.8%)	191050 (45.9%)
**Age group**				
<50 years	15276 (19.2%)	64989 (22.5%)	17188 (36.4%)	97453 (23.4%)
50-59 years	35764 (44.9%)	128925 (44.5%)	15331 (32.5%)	180020 (43.2%)
>59 years	28650 (36.0%)	95565 (33.0%)	14682 (31.1%)	138897 (33.4%)
**Blood pressure**				
0	63303 (79.4%)	120416 (41.6%)	1190 (2.5%)	184909 (44.4%)
2	14426 (18.1%)	131894 (45.6%)	22671 (48.0%)	168991 (40.6%)
3	1961 (2.5%)	37169 (12.8%)	23340 (49.4%)	62470 (15.0%)
Mean (SD)	0.436 (0.869)	1.30 (1.14)	2.44 (0.631)	1.26 (1.18)
**Blood sugar**				
0	8543 (10.7%)	8640 (3.0%)	192 (0.4%)	17375 (4.2%)
1	21459 (26.9%)	37802 (13.1%)	2063 (4.4%)	61324 (14.7%)
2	49688 (62.4%)	243037 (84.0%)	44946 (95.2%)	337671 (81.1%)
Mean (SD)	1.52 (0.681)	1.81 (0.462)	1.95 (0.239)	1.77 (0.511)
**Cholesterol**				
0	66340 (83.2%)	220229 (76.1%)	26032 (55.2%)	312601 (75.1%)
1	13350 (16.8%)	69250 (23.9%)	21169 (44.8%)	103769 (24.9%)
Mean (SD)	0.168 (0.373)	0.239 (0.427)	0.448 (0.497)	0.249 (0.433)
**BMI**				
0	38557 (48.4%)	60067 (20.8%)	1364 (2.9%)	99988 (24.0%)
1	32084 (40.3%)	135375 (46.8%)	12189 (25.8%)	179648 (43.1%)
2	9049 (11.4%)	94037 (32.5%)	33648 (71.3%)	136734 (32.8%)
Mean (SD)	0.630 (0.678)	1.12 (0.720)	1.68 (0.523)	1.09 (0.749)
**Nutrition**				
0	47288 (59.3%)	99355 (34.3%)	5662 (12.0%)	152305 (36.6%)
1	27025 (33.9%)	129438 (44.7%)	20461 (43.3%)	176924 (42.5%)
2	5377 (6.7%)	60686 (21.0%)	21078 (44.7%)	87141 (20.9%)
Mean (SD)	0.474 (0.620)	0.866 (0.731)	1.33 (0.678)	0.843 (0.742)
**Alcohol**				
0	49098 (61.6%)	126852 (43.8%)	8615 (18.3%)	184565 (44.3%)
1	22588 (28.3%)	110586 (38.2%)	21197 (44.9%)	154371 (37.1%)
2	8004 (10.0%)	52041 (18.0%)	17389 (36.8%)	77434 (18.6%)
Mean (SD)	0.484 (0.671)	0.742 (0.742)	1.19 (0.719)	0.743 (0.750)
**Smoking**				

	1st quintile (n = 79,690)	2nd-4th quintile (n = 288,479)	5th quintile (n = 47,201)	Overall (N = 416,370)
0	27702 (34.8%)	15450 (5.3%)	57 (0.1%)	43209 (10.4%)
3	51988 (65.2%)	274029 (94.7%)	47144 (99.9%)	373161 (89.6%)
Mean (SD)	1.96 (1.43)	2.84 (0.674)	3.00 (0.104)	2.69 (0.915)
**Aerobic activities**				
0	52632 (66.0%)	128718 (44.5%)	10879 (23.0%)	192229 (46.2%)
1	27058 (34.0%)	160761 (55.5%)	36322 (77.0%)	224141 (53.8%)
Mean (SD)	0.340 (0.474)	0.555 (0.497)	0.770 (0.421)	0.538 (0.499)
**Sleep**				
0	32704 (41.0%)	65008 (22.5%)	4589 (9.7%)	102301 (24.6%)
1	46986 (59.0%)	224471 (77.5%)	42612 (90.3%)	314069 (75.4%)
Mean (SD)	0.590 (0.492)	0.775 (0.417)	0.903 (0.296)	0.754 (0.431)
**Stress**				
0	33138 (41.6%)	71189 (24.6%)	6010 (12.7%)	110337 (26.5%)
1	46552 (58.4%)	218290 (75.4%)	41191 (87.3%)	306033 (73.5%)
Mean (SD)	0.584 (0.493)	0.754 (0.431)	0.873 (0.333)	0.735 (0.441)
**Social relationship**				
0	11643 (14.6%)	20892 (7.2%)	1706 (3.6%)	34241 (8.2%)
1	68047 (85.4%)	268587 (92.8%)	45495 (96.4%)	382129 (91.8%)
Mean (SD)	0.854 (0.353)	0.928 (0.259)	0.964 (0.187)	0.918 (0.275)
**Total Brain Care Score**				
Mean (SD)	8.03 (1.21)	11.9 (1.34)	15.5 (0.766)	11.6 (2.42)

Legend. Blood sugar levels were measured as hemoglobin A1c (HbA1C). The red meat score is based on beef, pork, and lamb/mutton consumption, in which an individual score was first assigned for each meat type ("Never" or "Less than once a week" with 0; "Once a week" or "2-4 times a week" with 1; and "5-6 times a week" or "Once or more daily" with 2); these were then summed, with a score of 1-2 dichotomized into 1 and less than 1 or more than 2 with a 0. Moderate activity includes physical activities such as carrying light loads or cycling; vigorous activity includes activities such as fast cycling, aerobics, or heavy lifting. BMI stands for Body Mass Index.

The table shows the individual components of the Brain Care Score (number and proportion of participants in each score and the overall mean) by quintile group of the total Brain Care Score. Participants that were categorised into the second, third and fourth quintiles were combined into one group. The total Brain Care Score is the sum of all component scores with a total observed range from 1 to 19.

Table S10. Cumulative incidence of late-life depression, and dementia or stroke or late-life depression at baseline by Brain Care Score quintile group

	Cumulative incidence of late-life depression (95% CI)	Cumulative incidence of the composite outcome (95% CI)
Overall	0.018 (0.018 - 0.019)	0.038 (0.037 - 0.038)
1st quintile (BCS: 1-9)	0.024 (0.023 - 0.026)	0.048 (0.046 - 0.05)
2nd-4th quintile (BCS: 10-14)	0.019 (0.018 - 0.019)	0.04 (0.039 - 0.04)
5th quintile (BCS: 15-19	0.013 (0.012 - 0.014)	0.026 (0.025 - 0.027)

Legend. BCS = Brain Care Score; 95% CI = 95% confidence interval.

Table S11. Coefficient table for late-life depression models in the GP data

	Incident late-life depression		
	**Overall**	**50-59 years**	**>59 years**
Per 5-point increase in Brain Care Score	0.60***	0.59***	0.62***
	(0.54 to 0.65)	(0.51 to 0.70)	(0.55 to 0.69)
Age	1.12***		
	(1.11 to 1.13)		
Sex: males	0.82***	1.01	0.78***
	(0.75 to 0.90)	(0.86 to 1.20)	(0.70 to 0.87)
Observations	192,468	64,615	80,974
Cases	2,037	597	1,440

Legend. Reference group: females (sex).
*p<0.05; **p<0.01; ***p<0.001

Table S12. Coefficient table for late-life depression Fine and Gray subdistribution hazard model

	Incident late-life depression			
	**Overall**	**<50 years**	**50-59 years**	**>59 years**
Per 5-point increase in Brain Care Score	0.69***	0.41***	0.66***	0.73***
	(0.65 to 0.72)	(0.27 to 0.64)	(0.61 to 0.72)	(NA to NA)
Age	0.59***			
	(0.56 to 0.62)			
Sex: males	1.09***	0.52***	0.63***	0.60***
	(1.08 to 1.09)	(0.32 to 0.84)	(0.58 to 0.69)	(NA to NA)
Observations	363,323	86,323	122,995	154,005

Legend. Reference group: females (sex).
*p<0.05; **p<0.01; ***p<0.001

Table S13. Coefficient table for composite event Fine and Gray subdistribution hazard model

	Incident dementia *or* stroke *or* late-life depression			
	**Overall**	**<50 years**	**50-59 years**	**>59 years**
Per 5-point increase in Brain Care Score	0.75***	0.62***	0.65***	0.80***
	(0.72 to 0.78)	(0.52 to 0.75)	(0.61 to 0.70)	(0.76 to 0.84)
Age	1.11***			
	(1.10 to 1.11)			
Sex: males	0.94***	1.39***	0.90 **	0.95***
	(0.91 to 0.97)	(1.16 to 1.66)	(0.84 to 0.96)	(0.91 to 0.99)
Observations	358,198	85,893	121,690	150,615

Legend. Reference group: females (sex).
*p<0.05; **p<0.01; ***p<0.001

Table S14. READ2 and READ3 codes used to ascertain depression in the General Practitioner subset

Description	**Cut-off (if relevant)**	**READ2**	**READ3**
Personal history of depression	-	1465.	1465.
Recurrent depression	-	E1137	E1137
Chronic depression	-	E2B1.	E2B1.
Depression: [episode, unspecified] or [NOS (& reactive)] or [depressive disorder NOS]	-	Eu32z	XE1Zb, Eu32z, XE1YC, XaB9J, XE1Zb
Agitated depression	-	7L1R0	X00SQ
Endogenous depression	-	E112.	X00SR
Endogenous depression first episode	-	E112.	X00SS
Moderate depression	-	1B17., E11.., Eu321	XaCIt
Severe depression		1B17., E11.., Eu32., Eu326	XaCIu
Patient advised about the management of depression	-	8CAa.	XaKEz
Endogenous depression – recurrent	-	E113.	XM1GC
Depression annual review	-	9H90.	XaK6d
Depression medication review	-	9H91.	XaK6e
HAD depression scale	>= 8[2]	388P., 388J.	XaIwf, XM0eK
DASS-Depression scale	>= 10[3]	388Z.	XaKaX
Beck DI 2 score	>= 9[4]	388g.	XaLLG
Geriatric depression scale	>= 5[5,6]	388K.	XM0df

Supplementary Figures

Fig. S1 | Flowchart for the General Practitioner data cohort

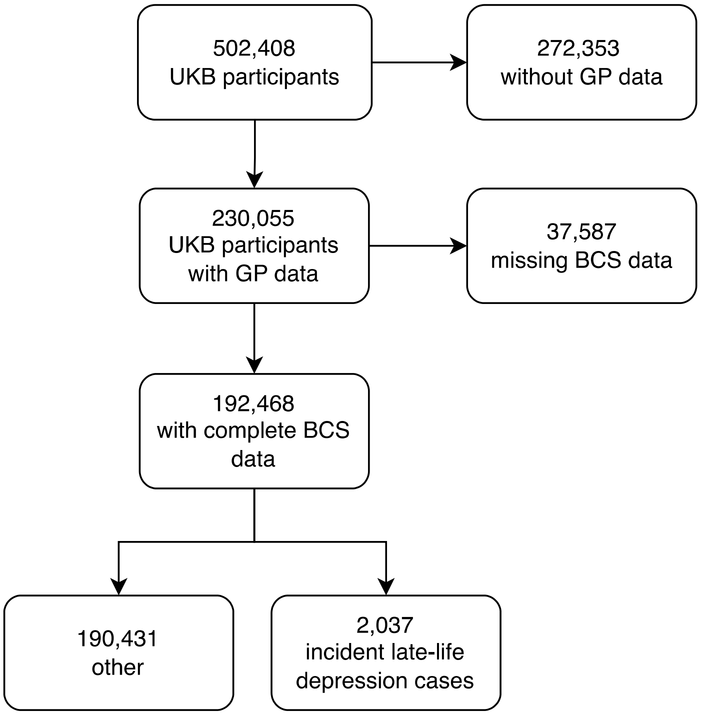

Fig. S2 | Cumulative incidence of late-life depression in the GP data cohort, stratified by Brain Care Score quintile group

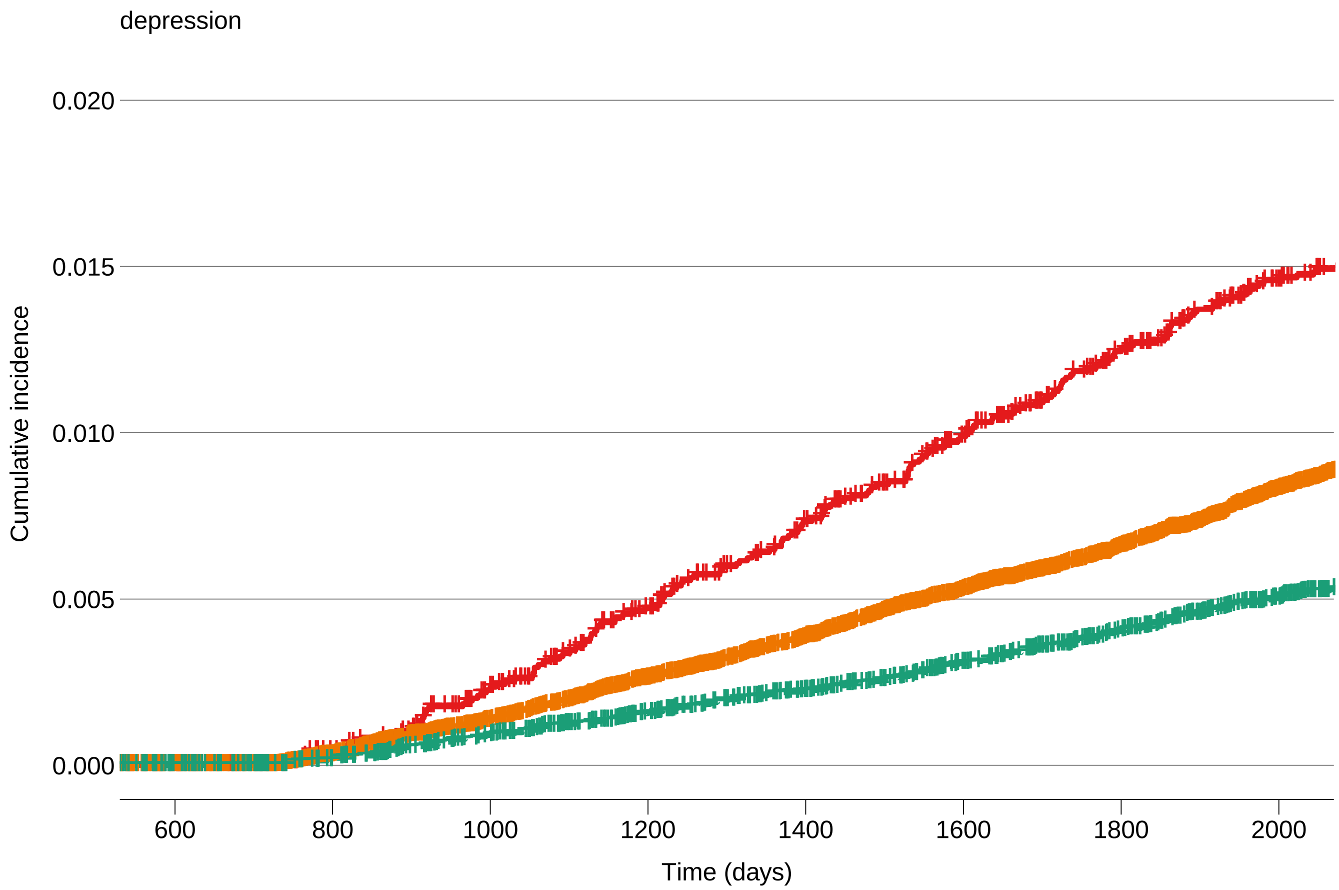

Fig. S3 | Schoenfeld residuals for proportional hazards assumption with incident late-life depression as outcome

a) Main model

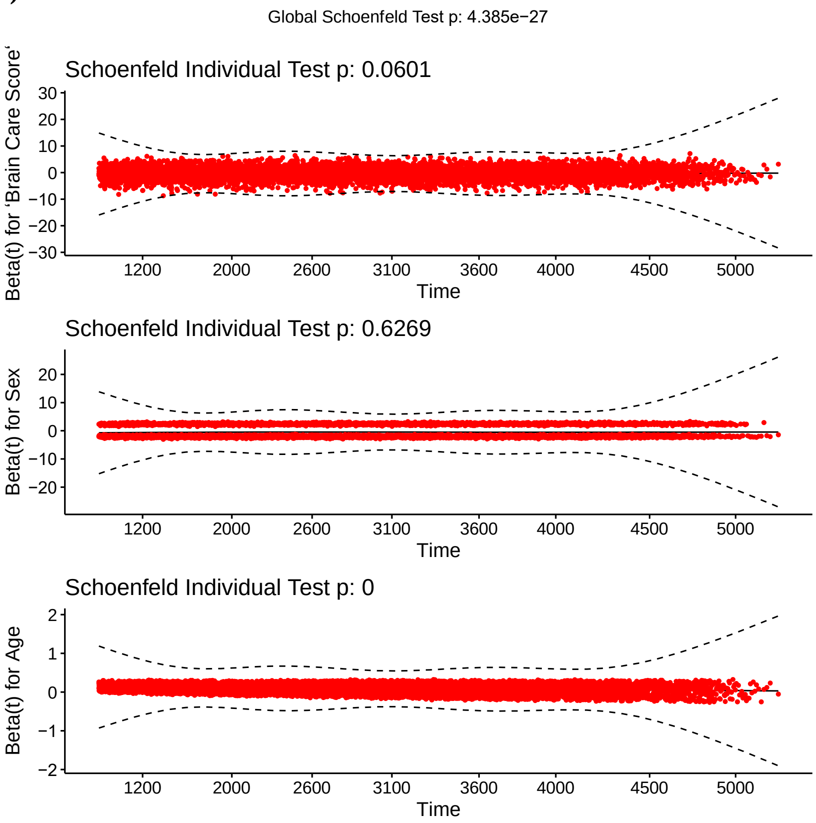

b) <50 years

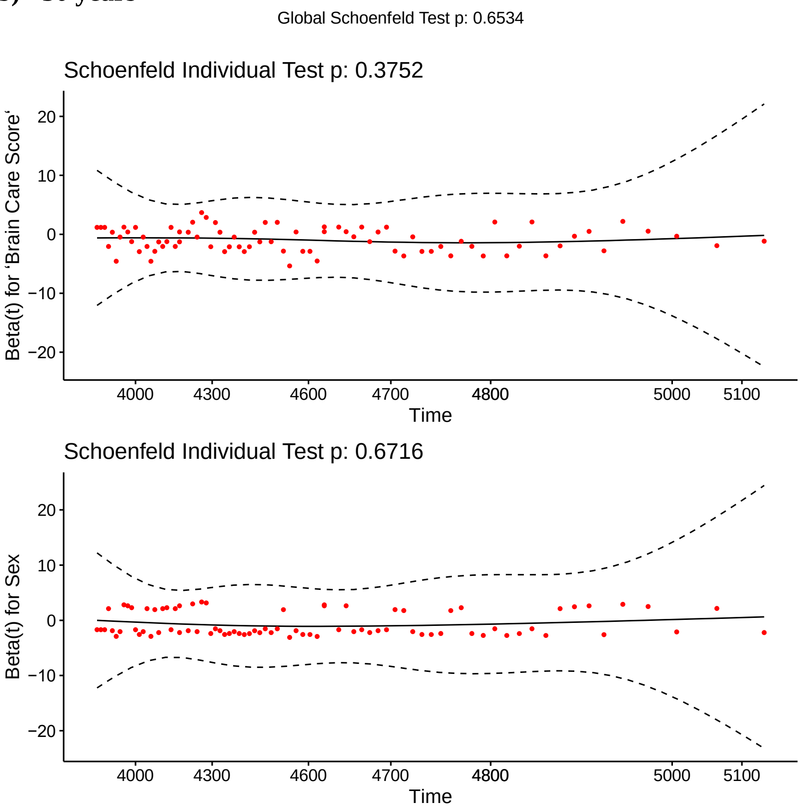

c) 50-59 years

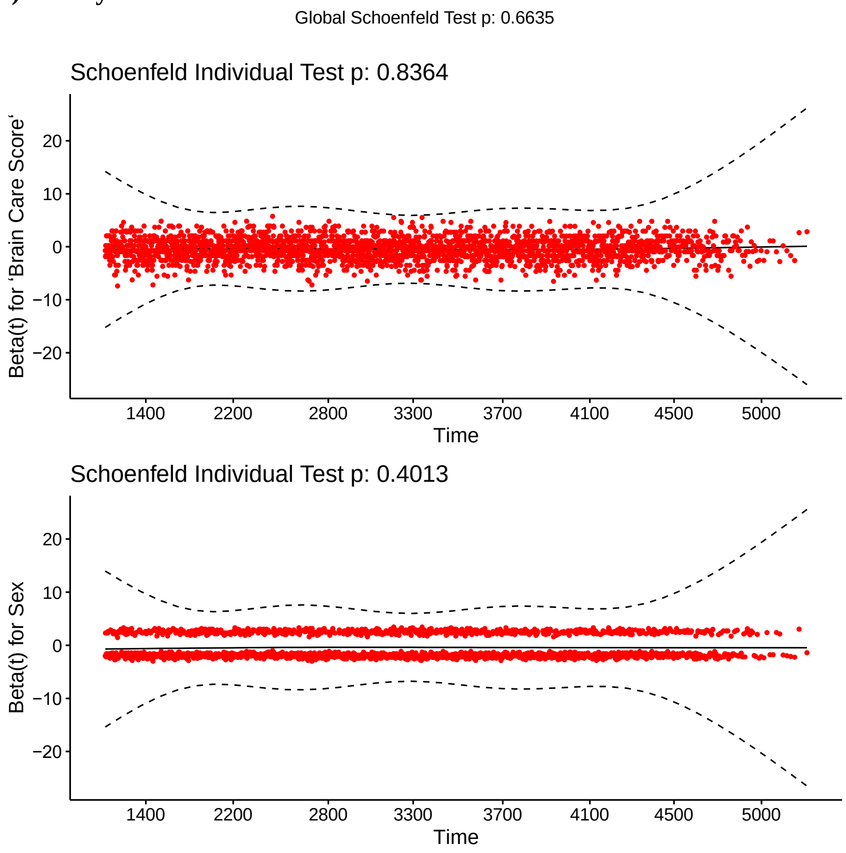

d) >59 years

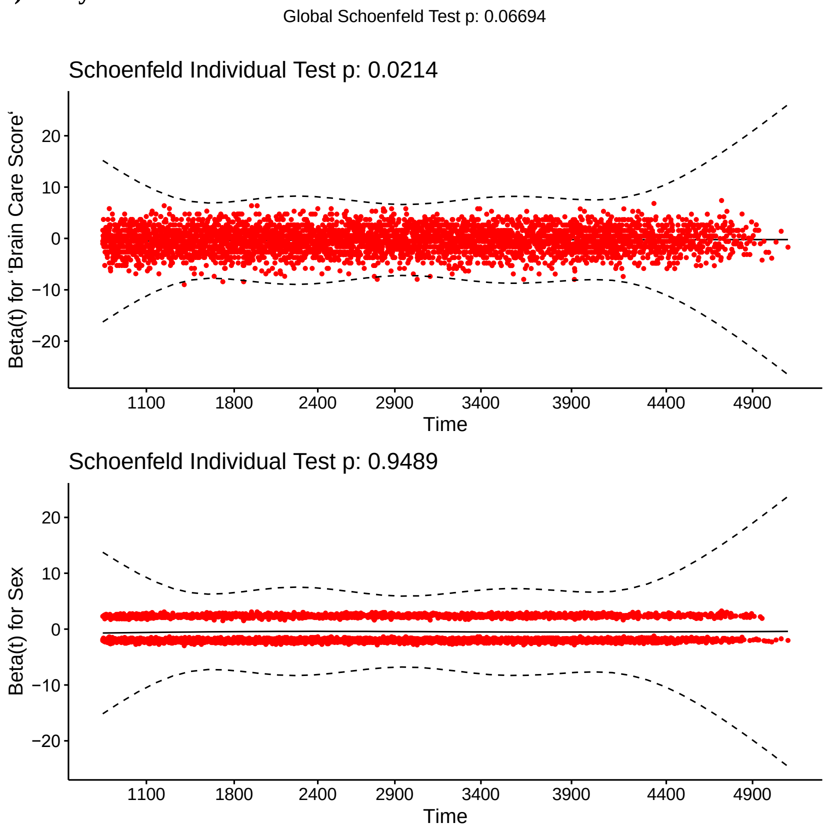

Fig. S4 | Schoenfeld residuals for proportional hazards assumption with incident dementia *or* stroke *or* late-life depression as outcome

a) Main model

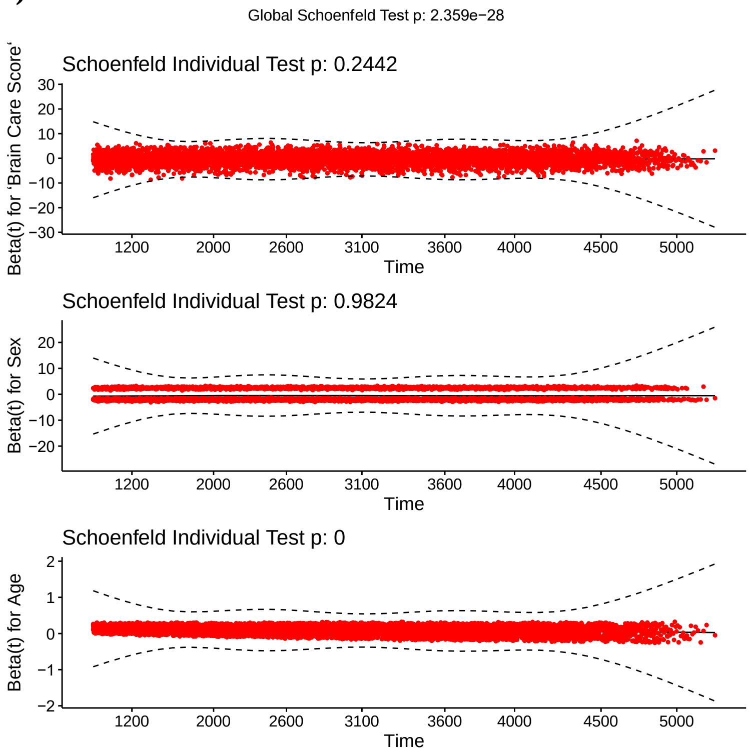

b) <50 years

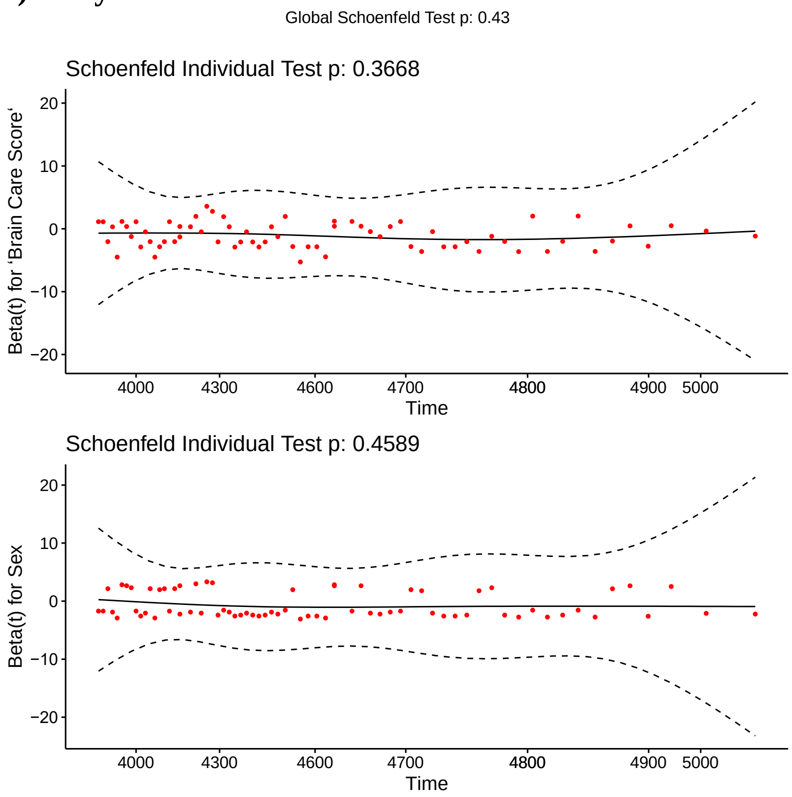

c) 50-59 years

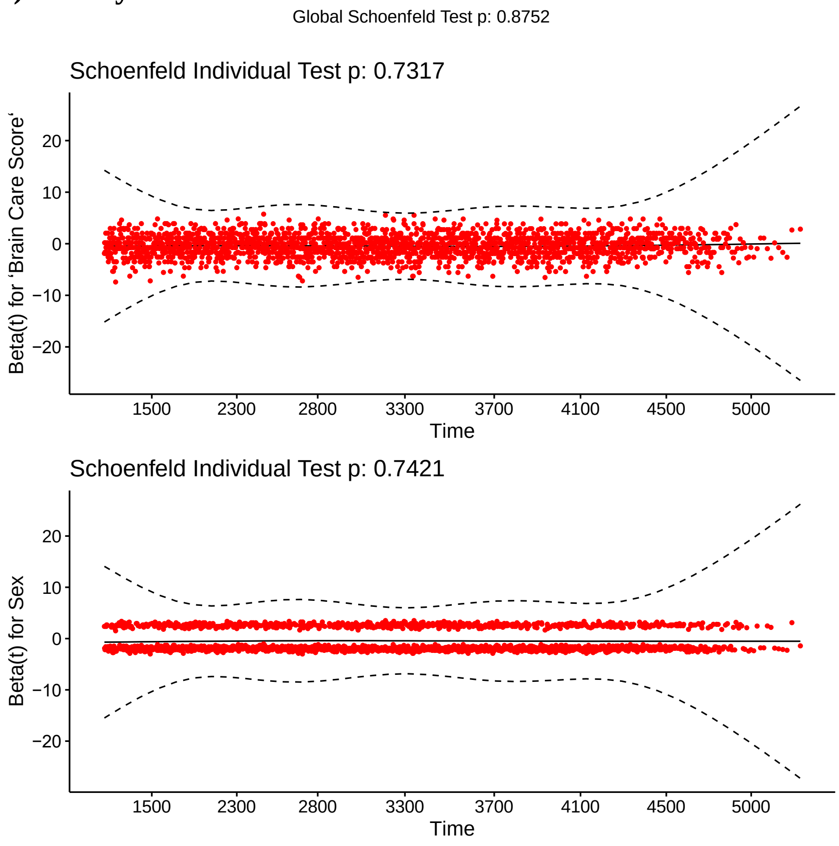

d) >59 years

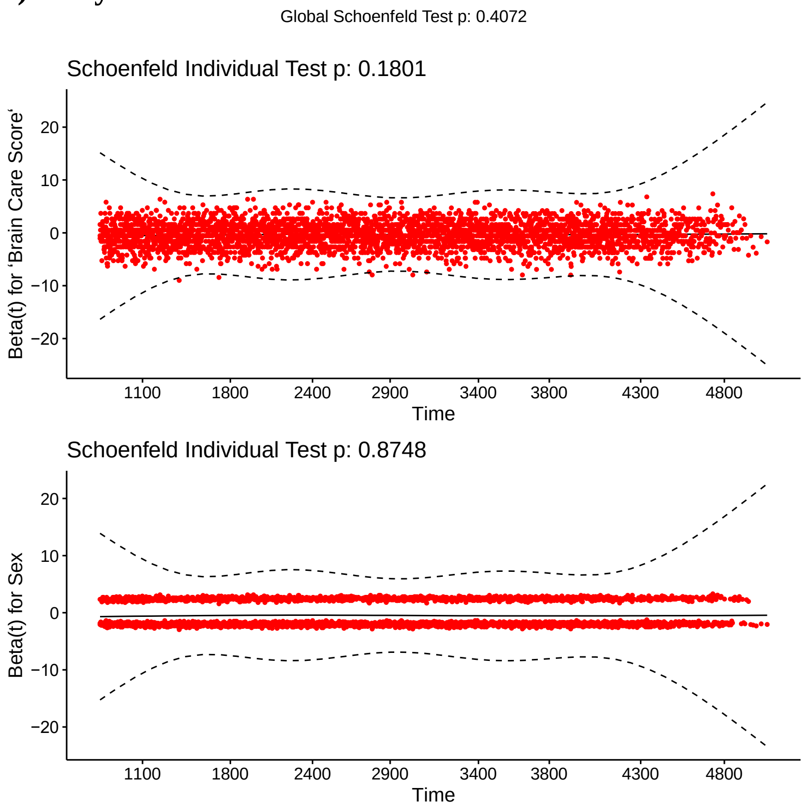

Bibliography

1. Zhou X h, Li C m, Yang Z. Improving interval estimation of binomial proportions. *Philos Trans R Soc Math Phys Eng Sci*. 2008;366(1874):2405-2418. doi:10.1098/rsta.2008.0037

2. Hansson M, Chotai J, Nordstöm A, Bodlund O. Comparison of two self-rating scales to detect depression: HADS and PHQ-9. *Br J Gen Pract*. 2009;59(566):e283-e288. doi:10.3399/bjgp09X454070

3. Peters L, Peters A, Andreopoulos E, Pollock N, Pande RL, Mochari-Greenberger H. Comparison of DASS-21, PHQ-8, and GAD-7 in a virtual behavioral health care setting. *Heliyon*. 2021;7(3):e06473. doi:10.1016/j.heliyon.2021.e06473

4. Beck AT, Steer RA, Brown G. Beck Depression Inventory–II. Published online 1996. doi:10.1037/t00742-000

5. Laudisio A, Antonelli Incalzi R, Gemma A, et al. Definition of a Geriatric Depression Scale cutoff based upon quality of life: a population-based study. *Int J Geriatr Psychiatry*. 2018;33(1):e58-e64. doi:10.1002/gps.4715

6. Yesavage JA, Brink TL, Rose TL, et al. Development and validation of a geriatric depression screening scale: a preliminary report. *J Psychiatr Res*. 1982;17(1):37-49. doi:10.1016/0022-3956(82)90033-4
